# Diversity of Arbuscular Mycorrhizal Fungi in Distinct Ecosystems of the North Caucasus, a Temperate Biodiversity Hotspot

**DOI:** 10.3390/jof10010011

**Published:** 2023-12-24

**Authors:** Andrey P. Yurkov, Alexey A. Kryukov, Anastasiia O. Gorbunova, Tatyana R. Kudriashova, Anastasia I. Kovalchuk, Anastasia I. Gorenkova, Ekaterina M. Bogdanova, Yuri V. Laktionov, Peter M. Zhurbenko, Yulia V. Mikhaylova, Roman K. Puzanskiy, Tatyana N. Bagrova, Oleg I. Yakhin, Alexander V. Rodionov, Maria F. Shishova

**Affiliations:** 1 Laboratory of Ecology of Symbiotic and Associative Rhizobacteria, All-Russia Research Institute for Agricultural Microbiology, Pushkin, 196608 St. Petersburg, Russia; aa.krukov@arriam.ru (A.A.K.); gorbunova.anastasia93@mail.ru (A.O.G.); tahacorfu@yandex.ru (T.R.K.); k.nastya4321@gmail.com (A.I.K.); nastya.gorenkova.2016@mail.ru (A.I.G.); bogdanova.ekaterina15@gmail.com (E.M.B.); laktionov@arriam.ru (Y.V.L.); 2 Graduate School of Biotechnology and Food Science, Peter the Great St. Petersburg Polytechnic University, 194064 St. Petersburg, Russia; 3 Faculty of Biology, St. Petersburg State University, 199034 St. Petersburg, Russia; mshishova@mail.ru; 4 Laboratory of Biosystematics and Cytology, Komarov Botanical Institute of the Russian Academy of Sciences, 197022 St. Petersburg, Russia; pj_28@mail.ru (P.M.Z.); ymikhaylova@binran.ru (Y.V.M.); avrodionov@mail.ru (A.V.R.); 5 Laboratory of Analytical Phytochemistry, Komarov Botanical Institute of the Russian Academy of Sciences, 197022 St. Petersburg, Russia; puzansky@yandex.ru; 6 Faculty of Ecology, Russian State Hydrometeorological University, 192007 St. Petersburg, Russia; tatyana-bagrova@mail.ru; 7 Institute of Biochemistry and Genetics, The Ufa Federal Research Center of the Russian Academy of Sciences, 450054 Ufa, Russia; yakhin@anrb.ru

**Keywords:** biodiversity, arbuscular mycorrhizal fungi, Glomeromycetes, Caucasus, ITS1, ITS2, hotspot

## Abstract

Background: Investigations that are focused on arbuscular mycorrhizal fungus (AMF) biodiversity is still limited. The analysis of the AMF taxa in the North Caucasus, a temperate biodiversity hotspot, used to be limited to the genus level. This study aimed to define the AMF biodiversity at the species level in the North Caucasus biotopes. Methods: The molecular genetic identification of fungi was carried out with ITS1 and ITS2 regions as barcodes via sequencing using Illumina MiSeq, the analysis of phylogenetic trees for individual genera, and searches for operational taxonomic units (OTUs) with identification at the species level. Sequences from MaarjAM and NCBI GenBank were used as references. Results: We analyzed >10 million reads in soil samples for three biotopes to estimate fungal biodiversity. Briefly, 50 AMF species belonging to 20 genera were registered. The total number of the AM fungus OTUs for the “Subalpine Meadow” biotope was 171/131, that for “Forest” was 117/60, and that for “River Valley” was 296/221 based on ITS1/ITS2 data. The total number of the AM fungus species (except for virtual taxa) for the “Subalpine Meadow” biotope was 24/19, that for “Forest” was 22/13, and that for “River Valley” was 28/24 based on ITS1/ITS2 data. Greater AMF diversity, as well as number of OTUs and species, in comparison with that of forest biotopes, characterized valley biotopes (disturbed ecosystems; grasslands). The correlation coefficient between “Percentage of annual plants” and “Glomeromycota total reads” r = 0.76 and 0.81 for ITS1 and ITS2, respectively, and the correlation coefficient between “Percentage of annual plants” and “OTUs number (for total species)” was r = 0.67 and 0.77 for ITS1 and ITS2, respectively. Conclusion: High AMF biodiversity for the river valley can be associated with a higher percentage of annual plants in these biotopes and the active development of restorative successional processes.

## 1. Introduction

One of the critical factors of species diversity conservation is the existence of biodiversity hotspots—regions with extremely high levels of species richness and endemism, with a high number of threatened species [1,2]. Researchers initially attributed hotspots to tropical forests [3], but with the intensive investigation of global biota, our world revealed many other hotspots. According to the last revision, there are 36 biodiversity hotspots [4]. The hotspot concept has been subjected to considerable criticism (summarized in [2]), mainly because the majority of studies in the field of hotspot biodiversity have only focused on plant and vertebrate species. In contrast, the diversity of invertebrates and fungi has not been studied enough [5]. The modern approach to biodiversity conservation should be more complex and include different aspects, like interactions between various organisms [2]. Arbuscular mycorrhizal fungi (AMF) can be important in addressing biodiversity conservation. AMF are microscopic soil fungi that form the most common type of plant–microbial symbiosis. Obligate symbiotic relationships with AMF characterize 71% of terrestrial plants, and only 7% of plants form inconsistent “nonmycorrhizal—mycorrhizal” associations [6]. Plants receive a number of benefits from this interaction: fungi improve the uptake of phosphorus, other nutrients, and water, increase resistance to pathogens, and protect plants from toxic heavy metals [7,8,9].

AMF diversity research in several tropical biodiversity hotspots has revealed significant species richness in these areas. For instance, 49 AMF species were identified in the Cerrado hotspot, Brazil [10]; 58 AMF species were found in the rhizosphere of plants from the Atlantic Forest [11]; and 72 species were discovered on a tropical mountain in Brazil [12]. While some AMF research has been conducted in tropical regions, only a few studies have investigated the AMF diversity in temperate hotspots.

One of the richest temperate regions in our world is the Caucasus [13]. Since 2000, it has been considered a biodiversity hotspot [1]. The Caucasus is a region on the isthmus between the Black and the Caspian Seas, on the border between Europe and Asia. The Caucasus ecoregion is shared by Russia (Karachay-Cherkess Republic, Republic of Dagestan, Republic of Ingushetia, Kabardino-Balkarian Republic, Republic of North Ossetia–Alania, and Chechen Republic), Georgia, Armenia, Azerbaijan, Turkey, and Iran. The Karachay-Cherkess Republic located on the northern macro slope of the Caucasus Mountains [14] is a promising region for studying biodiversity. It is characterized by a complex landscape, natural obstacles, frequent ecotope changes, mosaic vegetation cover, and phytocenoses diversity in the mountainous southern part of the region. The main waterway is the Kuban River, which only has left tributaries descending from the Caucasus Mountains (such as the Teberda River). Investigations into AMF diversity in biotopes of river valleys, forests, and alpine and subalpine meadows of Karachay-Cherkessia are very limited. While previous studies of the fungal biodiversity in the Caucasus have been carried out at the family [15] or at the genus level [16] via molecular genetic methods, no studies have addressed the AMF biodiversity in the Caucasus at the species level. Twelve AMF species were found in soil–root samples from southern Georgia [17]. However, this study only employed morphology-based identification, whereas molecular-based methods allow the identification of more taxa from the same soil and root samples than the morphological approach allows [18,19].

To assess the molecular diversity of AMF, researchers use the DNA-metabarcoding approach, including the high-throughput sequencing of a DNA barcode from the soil and root samples, followed by bioinformatics analysis. The critical point for fungal metabarcoding is the length of the obtained sequences. Therefore, high-throughput sequencing platforms can produce longer reads. Before 2020, mainly pyrosequencing (454-Roche) was employed [18,20,21,22]; now, Illumina MiSeq prevails in fungal metabarcoding studies [19,23,24,25]. Another promising platform is PacBio, which can obtain a sequence of 2.5 kb of rDNA in one read [26].

There are no consensus metabarcoding markers for AMF, but almost all today’s barcodes are parts of the rDNA cistron. In the last decade, the rDNA’s internal transcribed spacers (ITSs) have become the most common universal fungal metabarcoding marker [27]. Fragments of SSU (small sub-unit, or 18S) rRNA genes [18,22,24], ITS [21,28], and fragments of the LSU (large subunit, or 28S) rRNA gene [20,29] often serve as barcodes for AMF. For amplification of the SSU rRNA gene fragments, AMF-specific primer pairs are now in development (for example, [30]). In this case, this study’s goal is to observe not only AMF, but also the diversity of various fungi classes, ITS and D1/D2 regions were used [20,21]. For identification based on ITSs, universal eucaryotic primers or a combination of fungi-specific primers with universal primers are used [21]. 

DNA markers other than rDNA are rarely utilized as barcodes. The pilot experiment showed that glomalin, a heat shock protein homolog, is a good marker for AMF identification [31]. However, the major drawback of introducing new barcodes is the need for reference data. A reference database should contain a representative number of correctly identified taxa to identify species successfully. Almost 350 species of AMF have been correctly described according to the Botanical Nomenclature Code [32]. GenBank NCBI [33], the biggest public database, contains the glomalin data for only 24 species. In addition to glomalin, researchers and scientists used the following genes and regions as barcodes: (1) the mitochondrial cytochrome c oxidase 1, COX1 or COI [34,35]; (2) LSU of mitochondrial rRNA [36]; (3) I and II subunits of RNA polymerase II [37]; (4) H+-ATPase [38]; (5) *β*-tubulin [39]; (6) actin; (7) elongation factor 1-*α*, EF 1-*α*; (8) phosphate transporters; (9) RNA polymerase II subunits (RPB1 and less RPB2) [40,41]. Most of these markers have not been widely used due to the low nucleotide polymorphism between closely related AMF species. Perhaps the *rpb1* gene can become the second barcode in quality after the 18S-ITS1-5.8S-ITS2-28S region, as *rpb1* was successfully used to identify the AMF species [41,42].

The systematics of AMF is quite complex and controversial. There are many synonyms; many genera were taxonomically revised, but not all AMF species names were effectively published after the revisions according to the Botanical Nomenclature Code. Many taxa found in soil samples are described only by the DNA barcode and have no proper scientific names. AMF form the Glomeromycota phylum and are classified into one class, Glomeromycetes, and four orders [32]. However, according to Castillo et al. [43], AMF form the Glomeromycota phylum and are classified into three classes and five orders. According to the database curated by SYMPLANTA (Symbiosis and Plant-Microbe Association Research Laboratory), there are four orders, 12 families, 44 genera, and 443 species of AMF fungi [32]. The GenBank NCBI represents various genera unevenly. The database contains most species from the *Claroideoglomus*, *Dominikia*, *Septoglomus*, *Corymbiglomus*, and *Diversispora* genera. Poorly represented genera include *Glomus sensu lato*, *Scutellospora*, *Paraglomus*, and *Pacispora*. Thus, the GenBank database is not complete and lacks many species. Due to the problem of precisely identifying AMF species, dozens of virtual taxa were obtained [19,22,23,44]. Alternatives for the GenBank NCBI database include special reference databases. One of the central curated databases is MaarjAM; it primarily includes the SSU rRNA gene-based barcodes and the checked metadata for each entry [45]. This database is regularly updated and contains data for different barcodes [46]. The most accurate assessment of the biodiversity of AMF (Glomeromycetes) and other fungal classes is their identification using rDNA ITSs as universal barcodes.

One of the most important directions of modern studies is to identify the key factors causing AMF biodiversity. Temperature conditions, agrochemical properties of soils (including the pH level), usage of meadows as grassland, and changes in plant communities in the succession process are examples of them [47,48,49,50]. According to Horn et al. [51], biotic factors have a more significant impact on the composition of AMF communities than abiotic factors. The development of succession from a meadow to a forest, accompanied by an increase in the proportion of perennial plants in the ecosystem, maybe the reason for a decrease in the abundance of AMF [52,53]. According to Torrecillas et al., annual plants had a higher AMF diversity [54]. Nevertheless, there is currently no convincing and clear evidence that identifies a key factor responsible for the high species diversity of AMF. Another important factor is altitude. Studies show that a subalpine meadow in the Head Mountain, Inner Mongolia Autonomous Region, located at an altitude of over 2500 m above sea level, exhibited the most diverse AMF population [50]. On the other hand, in the Southern-Central zone of Chile, the “natural grassland” ecosystem exhibited the greatest diversity of AMF species at a significantly lower altitude (<500 m above sea level) [43]. Perhaps there are no direct correlations between altitude and AMF biodiversity [51,55,56,57,58]. Further elucidation of the factors contributing to the increase in AMF species diversity is required. Thus, it is essential to test the supposition that the biodiversity of AM fungi may be primarily related to such factors as soil pH, phosphorus content, altitude above sea level, and plant species richness. This study aims to detect the AMF hotspots and define the AMF biodiversity in rhizospheric soils of different biotopes in the North Caucasus (such as a subalpine meadow, a forest and a river valley). The research objectives are: (1) to sequence fungal DNA samples from the rhizospheric soil using Illumina MiSeq and ITS1 and ITS2 regions as barcodes; (2) to assess the species diversity of AM fungi; and (3) to assess the key relationships between parameters of the biodiversity of AM fungi and plant species richness, altitude for trial plots, and agrochemical soil parameters.

## 2. Materials and Methods

### 2.1. The Characteristics of the Stationary Trial Plots

The characteristics of Stationary Trial Plots (STPs, study areas, sample plots), as well as the soils in which the species diversity of AMF was determined are presented in Table 1 and Table S1. We analyzed the following STPs in different altitudes: (1) in subalpine meadow biotopes—STP 1 (subalpine meadow-4, Malaya Hatipara ridge, 2437 m above sea level), STP 3 (subalpine meadow-3, Malaya Hatipara ridge, 2401 m above sea level), and STP 4 (subalpine meadow-2, Malaya Hatipara ridge, 2186 m above sea level); (2) in forest biotopes—STP 7 (fir forest-3, Malaya Hatipara mountain, 1900 m above sea level), STP 8 (pine forest-3, Malaya Hatipara mountain, 1890 m above sea level), and STP 9 (mixed forest near the Bolshaya Hatipara river, Bolshaya Hatipara mountain, 1507 m above sea level); (3) in river valley biotopes—STP 11 (grassland in the valley of the Teberda river, Teberda town, 1342 m above sea level), STP 12 (grassland in the valley of the Teberda River, the border of the New Teberda Village, 1026 m above sea level), and STP 13 (grassland in the valley of the Kuban River, Ordzhonikidzevsky village, 795 m above sea level). The trial area was 10 m × 10 m. A botanical survey of test sites, STPs located in undisturbed ecosystems of the North Caucasus, the Teberdinsky National Park, and adjacent territories, was carried out using Zernov’s determinants ([14,59]; see Table S2). In each STP, the soil samples were collected for molecular genetic analysis of fungal diversity from the upper 0–5 cm horizon of the soil without litter according to [16], and the samples of the upper horizon of the soil without litter were collected for agrochemical analysis according to [60]. The total phosphorus content (P_total_, %) via Ginzburg and Shcheglova’s method, the content of phosphorus available for plant nutrition (Pi, mg/kg) via the Truog method, the total nitrogen content (N_total_, %) via the Kjeldahl method; and pH_KCl_, the sum of fractions < 0.01 mm, and soil type via common methods were determined ([61]; see Table S1).

### 2.2. Molecular Genetic Identification of Fungi

#### 2.2.1. Sampling and Molecular Analysis

For the molecular genetic identification of AMF, the authors used their optimized sampling technique for Illumina MiSeq sequencing. In total, we derived 10 samples from each STP. The samples were frozen and transported in 50 mL tubes in liquid nitrogen. We took samples of rhizospheric soil containing mycelium and AMF spores for identification (microscopic verification). To isolate DNA, 0.2 g of frozen soil and 0.5 g of garnet sand were put into a 2 mL test Eppendorf tube for mechanical grinding (degradation) of the material. After that, 700 µL of CTAB buffer was added to the heated test tube with soil (2% CTAB; 1.4 M NaCl; 20 mM EDTA; 100 mM Tris-HCl pH = 8.0). Initially, the material was homogenized in a buffer with garnet sand for 15 min on a vortex. After that, the test tubes were shaken every 15 min for 1 h at +65 °C using a laboratory vortex shaker (Biosan, Riga, Latvija). After thermal, chemical, and mechanical treatment, the samples were centrifuged for 5 min, and the supernatant was transferred to new test tubes. The DNA was additionally washed off the soil residues with 500 μL of water by shaking soil with water in the vortex for 5 min. After additional centrifugation, the second supernatant was combined with the first one. The obtained DNA was purified via double extraction with an equal volume of chloroform. A DNA supernatant was selected each time after centrifugation (10 min at 14,000 rpm, Eppendorf, Hamburg, Germany). Then, the DNA was deposited with 2/3 V isopropanol with 0.4 M NaCl, and the precipitate was washed with 70% ethyl alcohol; the precipitate was dried (until the alcohol was completely removed) for 3 min and then dissolved in water [62,63]. After that, the DNA was purified with a Qiagen Gel Extraction Kit (Germany, Düsseldorf). Purification on columns or with silicon oxide after isolation of a DNA fragment on an agarose gel after electrophoresis does not eliminate various PCR-inhibiting impurities. Therefore, an additional measure could be the dilution of DNA samples before amplification by 100 times with water (accompanied by diligent mixing) to reduce the effect on PCR of inhibitors from the soil, including humic acids. 

The purified DNA was used for nested PCR marker regions ITS1 and ITS2 with universal primers. The primers were synthesized in Evrogen, Russia, with the 5′-TCGTCGGCAGCGTCAGATGTGTATAAGAGACAG-3′ adapter for direct primers and the 5′-GTCTCGTGGGCTCGGAGATGTGTATAAGAGACAG-3′ adapter for reverse primers for Illumina MiSeq, such as the ITS5 (5′-GGAAGTAAAAGTCGTAACAAGG-3′) primer and the reverse primer ITS-2RK (5′-CGTTCAAAGATTCGATGATTCAC-3′) modified by the authors for amplification of the ITS1 region, as well as ITS3 (5′-GCATCGATGAAGAACGCAGC-3′) primers and reverse ITS4 (5′-TCCTCCGCTTATTGATATGC-3′) for amplification of the ITS2 region. PCR: (1) PCR for the long fragment (ITS1 + ITS2 regions) with the ITS5 and ITS4 primers; the thermal cycling conditions were 5 min of initial denaturation and polymerase activation at 95 °C; 35 cycles of 20 s denaturation at 95 °C, 20 s of annealing at 58 °C and 40 s of elongation at 72 °C; and final elongation at 72 °C for 10 min; (2) verification of PCR with gel electrophoresis, DNA dilution by 1000 times; (3) 35 PCR cycles into short fragments (separately for ITS1 and ITS2 regions); repetition of the program with primers for nested PCR; and (4) quality control with gel electrophoresis, with the PCR products for ITS1 and ITS2 combined for each sample and purified on magnetic particles using an AMPure XP (Beckman Coulter, Brea, CA, USA).

The amplicon libraries were sequenced on an Illumina MiSeq device using a set of reagents from a MiSeq^®^ Reagent Kit v3 (600-cycle) with two-way reading (2 × 300 bp) (“Illumina, Inc.”, San Diego, CA, USA). The identified sequences were processed using Illumina software v2.6 (“Illumina, Inc.”, San Diego, CA, USA). Because of sequencing on the Illumina MiSeq platform, FASTQ sequences were obtained from forward and reverse primers. This format includes sequence records and reading quality indicators (quality score) for each nucleotide position. 

#### 2.2.2. Bioinformatics

We uploaded the Illumina data (FASTQ sequences) to the NCBI database; they are now available in the base as the PRJNA646244 bioproject [64]. Data processing was performed with USEARCH [65]. The forward and reverse reads were merged using the “fastq_mergepairs” command; sequences shorter than 120 bp were removed. The resulting reads constituted a pool of a single FASTQ file. Further processing to OTUs with a 97% radius was carried out in two steps. First, sequences from the MaarjAM database [45] were used as OTU centroids to map the obtained sequences in FASTQ format. Secondly, the sequences not mapped to the MaarjAM database (with a 97% threshold) were processed to OTUs using the UPARSE algorithm [66], where the most abundant sequences are selected as centroids. During this step, regions corresponding to primers were removed, and sequences were quality-filtered with the “fastq_maxee” parameter value of 1. The “cluster_otus” command removed the chimeric sequences during OTU construction; residual singletons were also removed. We used both types of centroids to construct the OTU table. The high genetic polymorphism observed in the internal transcribed spacers (ITSs) of AMF [67] makes it difficult to identify many AMF species accurately [33]. Consequently, an increasing number of virtual taxa (VT) are being added to databases [19,22,44]. For effective assessment of AMF diversity, Operational Taxonomic Units (OTUs) is recommended instead of Amplicon Sequence Variants (ASVs) or Zero-radius OTUs (ZOTUs) for the ITS1 and ITS2 AMF regions. For taxonomic annotation of the newly obtained OTUs, we used the MaarjAM database, as well as the established local reference database consisting of sequences of fungi of the Glomeromycetes class from the NCBI database. The annotation was performed using the “usearch_global” command. The coding regions of rRNA were trimmed, leaving the spacer sequences for further processing. Primary clustering was performed by constructing an alignment-free tree using the neighbor-joining (NJ) method in the paHMM program [68]. Based on this tree, sequence clusters that could be aligned properly were selected. These alignments were supplemented with reference sequences annotated at the species level and were used to construct trees using the maximum likelihood (ML) method in the IQ-TREE [69]. Fungi annotation at the phylum level was based on the UTAX reference dataset version 27.10.2022 [70].

The general algorithm for further identification is shown in Figure S1. The alignment of ITS sequences by genera was most effective since the inter-genus variability in ITS for arbuscular mycorrhiza (AM) fungi is too high and most often did not allow for reliable alignment of sequences, and this is the most significant factor for the construction of reliable phylogenetic trees. With the help of the Mega 7 software package [71], the sequences combined by genera into separate files were aligned automatically, and then a manual revision of the alignment was carried out. After manual refinement of the alignment and the BLAST procedure in the NCBI GenBank, some sequences were rejected as not belonging to this genus (rarely, to a taxon of a higher rank), due to the inability to align them within the ITS regions under study. During manual alignment, species-specific mutations were also identified, and their analysis provided additional information about the sequence similarities. In some cases, this was the only way to separate sequences of two close species, vital for the identification of species from the *Rhizophagus* genus. After that, phylogenetic trees were reconstructed in the Mega 7 software package. The resulting trees were used to refine the identification at the species level. Sequences clustered together to form a separate clade were identified as sequences belonging to the same taxon. If the resulting clade was found to include reference sequences from the GenBank belonging to the same species, such sequences were assigned to a particular species. To clarify the species affiliation, we also used information on the length of phylogenetic branches as well as *p*-distances calculated by us using the NCBI database for reference sequences (Table S3), bootstrap indexes, and the presence or absence of species-specific nucleotide substitutions (e.g., [72]). In complex cases, individual sequences were also identified considering the closest sequences based on the results of manual BLAST analysis in the NCBI GenBank, paying attention primarily to the year of obtaining the sequence presented in the GenBank. If the obtained sequences were grouped into a clade but not grouped with reference sequences, then they were assigned to a VT of the species level. If a VT was not clustered on a tree with other sequences, forming a well-separated clade and had low *p*-distance compared to others, then it was identified as VT of the genus level or higher. If multiple sequences were present in such VT, they were excluded from the analysis to avoid biases in estimating biodiversity, as it was difficult to determine the number of species they represented.

#### 2.2.3. Assessment of Biodiversity Indices

The Hill numbers were evaluated using the iNEXT package [73] for the R environment to assess the taxonomic diversity [74]. After that, the species richness (diversity order *q* = 0), Shannon diversity (*q* = 1), and Simpson diversity (*q* = 2) were calculated. To overcome the limitations associated with different sample sizes, the Hill numbers were also extrapolated to a sample tending to infinity (estimated asymptotic diversities). A 95% confidence interval was calculated for each extrapolated index.

#### 2.2.4. Statistics

The statistical analysis was processed in the R language environment 4.1.0 [74]. The *total-sum scaling* method normalized the data and OTUs for fungal classes. The analysis of variance (ANOVA) was used to assess the statistical significance (*p* < 0.05) of the differences. Principal component analysis (PCA) was applied using the *pcaMethods* package [75]. For unsupervised dimension reduction, multidimensional scaling (MDS) was applied. Spearman’s distances (1-*ρ*) and MDS were used with *stats* [74]. An analysis of similarities (ANOSIM) [76] was made with the vegan package [77], and Spearman’s distance (1-rho) was used. Hierarchical clustering was carried out using the *dendextend* package [78]. Spearman’s distances and the Ward method for cluster agglomeration were used. The packages of *ggplot2* [79] and a Venn diagram [80] were used to plot the graphs.

## 3. Results

### 3.1. Analysis of Fungal Sequences

A total of the fungi sequences in the ITS1 and ITS2 regions amounted to >10 million (Table 2). The average read depth for STPs of meadows, forests, and river valleys (pastures) exceeded 1.1 million; after filtering (merged reads after length trim), it became 376,000 reads (Table 2). The number of AM fungi reads according to ITS1 varied widely, the average for the nine STPs was 1774 AMF reads per STP. The Illumina MiSeq sequencing analysis results show that across the sequences, >62% of the reads (or >90% of OTUs) belong to the fungal sequences. Meanwhile, the number of reads for AMF varied more widely for STP in ITS2 than in ITS1. On average, there were 722 AMF reads per STP in the ITS2 region for the nine STPs. Thus, on average, the number of AMF rows in the ITS2 region was 2.5 times lower than in the ITS1 region. The highest number of AMF reads was also detected in samples STP 11 and STP 13, related to the river valley, and the lowest in samples STP 7 and STP 8, related to forest biotopes.

It is common practice to remove singletons in studies, as the analyzed objects often account for 50% of all reads. Nevertheless, the rows of AM fungi usually represent only 1–2% of all reads, so the analysis of singletons can contain important details on their diversity. On the one hand, singletons often contain chimeric sequences and can be eliminated at the stage of manual alignment. On the other hand, the exclusion of singletons would lead to a significant loss of true fungal species. The application of public database sequences as OTU centroids has provided several advantages. First, using the same centroids for OTU clustering in different experiments made it possible to compare the results better. Secondly, the singletons in the data were successfully mapped with the known centroids and were not lost at the data processing stages. Therefore, using the MaarjAM database to build OTUs turned out to be reasonable; this approach made saving about 15% of reliable singletons in the final data possible.

The results show that the number of AMF OTUs for the river valley biotopes was 2.5 times higher than for the mountain forest biotopes and 1.7 times higher than for the subalpine meadow biotopes in the ITS1 region. The number of OTUs for meadow biotopes was also higher than for forest biotopes (by 1.5 times), according to the ITS1 region. According to the ITS2 region, the number of OTUs for the river valley biotopes was 3.7 times higher than for the forest biotopes and 1.7 times higher than for the subalpine meadow biotopes. The number of OTUs for meadow biotopes was also higher than for forest biotopes (2.2 times), according to the ITS2 region. Summing up the results of the analysis, we can assume that the biotopes of pastures in the river valley may have the most remarkable diversity of taxa. In contrast, the least diversity characterizes the forest biotopes. Sample representation in low-dimensional space revealed from MDS (Figure S2) and score plots from PCA of the out profiles (Figure S3) confirmed the differences between the three studied biotopes.

### 3.2. Taxonomic Composition of Fungal Phyla and Classes in Soil Samples from the River Valley, Subalpine Meadow, and Forest

The number and relative abundance (%) of fungal OTUs at the phylum level were assessed to identify the objective relative abundance of AMF reads and the general structure of fungal communities in the studied ecotopes (Figure S4). The most common fungal phyla were Ascomycota, Basidiomycota, Glomeromycota, Rozellomycota, Mortierellomycota, Chytridiomycota, Mucoromycota (Figure S4). Kickxellomycota, and Olpidiomycota, and Zoopagomycota accounted for less than 1% (“Others” in Figure S4). AM fungi are included in the phylum Glomeromycota, a monophyletic group with one class, Glomeromycetes. Glomeromycota had a significantly higher relative abundance of OTUs in soil from disturbed ecosystems of pastures in river valleys than in soil from forests and subalpine meadows and had a significantly lower relative abundance of OTUs in soil from forest (*p* < 0.05; Student’s *t*-test). The abundance of ectomycorrhizal fungi (Ascomycota, Basidiomycota) in the analyzed biotopes had no significant differences, except for a reduced level (*p* < 0.05) of Basidiomycota for river valleys. Other fungal phyla did not simultaneously have substantial differences in biotopes, like Glomeromycota.

The following classes of fungi (see Figure S5) were identified in soil samples by ITS1 and ITS2 regions according to the proportion of OTUs for the biotopes of the subalpine meadow, forest biotopes, and pasture biotopes in the river valley. A total of nine fungal classes accounting for at least 2% were found in soil samples from the subalpine meadow, forest, and river valley (Figure S5). The results show that the same trend can be traced as in the analysis of phyla. With regard to Ascomycetes, we found that Sordariomycetes, Dothideomycetes, and Pezizomycetes were more common in the river valley, whereas Leotiomycetes was more common in the forest biotope. AM fungi (Glomeromycetes) were also well represented in soil samples from the river valley, but the difference between the valley and other biotopes was astonishing.

Thus, the results show a significant proportion of AMF (the Glomeromycetes class) in the total number of sequenced samples attributed to the Fungi kingdom, especially for the river valley. In different STPs, 0.1–6.9% of all reads and 5–11% of OTUs were identified as AMF via ITS1 sequencing. Meanwhile, 0.01–1.0% of all reads and 1–5% of OTUs were identified as AMF via ITS2 sequencing. The number of OTUs from Glomeromycetes in the river valley was significantly (*p* < 0.05) larger than in the subalpine meadow, and the OTU number in the subalpine meadow was significantly (*p* < 0.05) more than in the forest (Table 2). Thus, we can expect the greatest diversity of AMF species in the biotopes of the river valley pastures (STP 11, STP 12, and STP 13).

### 3.3. Identification of AMF Species in the River Valley, Subalpine Meadow, and Forest Biotopes

**Figure 1:**
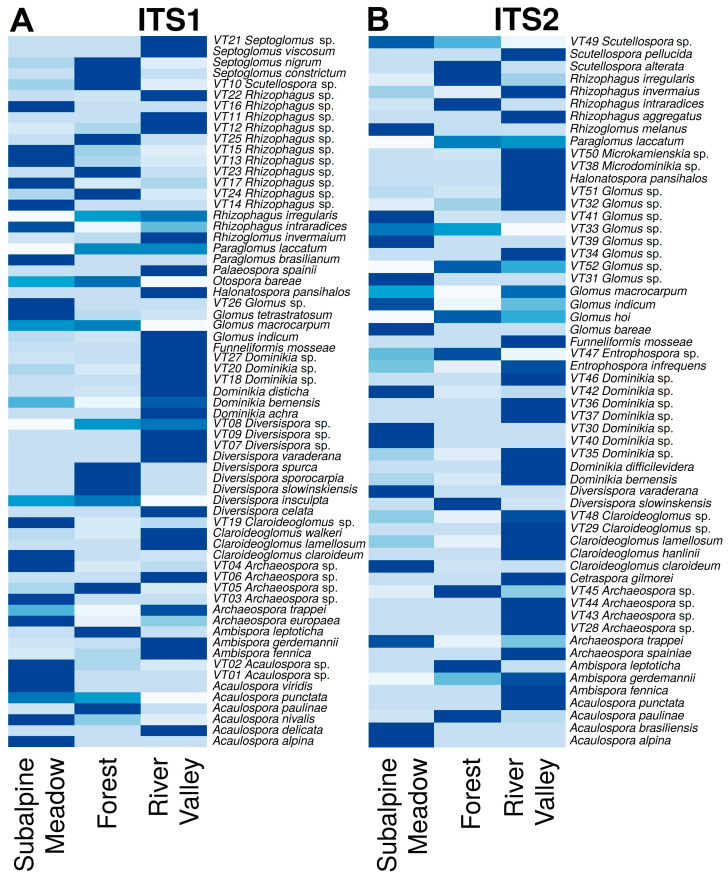
Heat map: frequency of occurrence of identified species in the ITS1 (**A**) and ITS2 (**B**) regions in three biotopes: subalpine meadow, forest, and river valley (calculated by the number of reads). The dark blue color on the heat map indicates a higher number of reads, and the light blue color indicates a lower number of reads.

Intraspecific polymorphism is challenging to assess due to the lack and heterogeneity of data in the NCBI GenBank database. To resolve this issue, we focused on the intra-genus *p*-distance (Table S3) calculated from the NCBI database using complex (remote from the main species clusters) sequences in the samples. Following the developed algorithm (Figure S1), phylogenetic trees were analyzed for more accurate identification of AMF species, known species were identified, as well as virtual taxa of the species level; an example of maximum likelihood (ML) phylogenetic trees for the Ambispora and Acaulospora genera is shown in Figures S6–S9. The virtual taxa at the species level were defined. VTs made up a significant part of the detected sequences. The construction of phylogenetic trees for all AMF genera, based on the analysis of ITS1 and ITS2 individual regions, resulted in the identification of several AMFs available in the analyzed soil samples. Figure 1 shows heat maps with the frequency of occurrence of identified AMF species by the number of reads for the ITS1 and ITS2 regions.

The analysis of phylogenetic trees revealed that a significant number (>42%) of the detected and identified AMF reads to the species level are virtual taxa. Almost the same proportion (~38%) was made up of AMF reads identified to the level of genus, family, and higher rank of taxa (it was not included in the performed analysis because it cannot characterize species diversity). The assumption is that the AMF discovered in the North Caucasus are not fully annotated by genetic methods. Only the construction of trees separately by genera (or by groups of close genera) is supposed to identify the taxa at the species level with a high variability of genetic markers. The following AMF taxa defined up to the species level were reliably identified in STPs in three different biotopes by constructing phylogenetic trees. The following known species were identified for ITS1 (see Table S4):

- Subalpine meadow (Acaulospora alpina, Ac. nivalis, Ac. nivalis, Ac. paulinae, Ac. punctata, Ac. viridis, Ambispora gerdemannii, Am. leptoticha, Archaeospora europaea, Ar. trappei, Claroideoglomus claroideum, Cl. walkeri, Diversispora insculpta, Dominikia bernensis, Do. disticha, Glomus indicum, G. macrocarpum, G. tetrastratosum, Otospora bareae, Paraglomus brasilianum, Paraglomus laccatum, Rhizophagus intraradices, Rhizophagus irregularis, Septoglomus constrictum, S. nigrum), or 24 species from 12 genera, as well as 19 virtual taxa at the species level;
- Forest (Acaulospora nivalis, Ac. paulinae, Ac. punctata, Ambispora fennica, Am. gerdemannii, Am. leptoticha, Claroideoglomus claroideum, Diversispora insculpta, Di. slowinskiensis, Di. sporocarpia, Di. spurca, Dominikia bernensis, Glomus indicum, G. macrocarpum, G. tetrastratosum, Otospora bareae, Paraglomus laccatum, Rhizoglomus invermaium, Rhizophagus intraradices, Rhizophagus irregularis, Septoglomus constrictum, S. nigrum), or 22 species from 11 genera, as well as 12 virtual taxa at the species level;
- River valley (Acaulospora delicata, Ac. paulinae, Ambispora fennica, Am. gerdemannii, Archaeospora europaea, Ar. trappei, Claroideoglomus claroideum, Cl. lamellosum, Cl. walkeri, Diversispora celata, Di. varaderana, Dominikia achra, Do. bernensis, Do. disticha, Funneliformis mosseae, Glomus indicum, G. macrocarpum, G. tetrastratosum, Halonatospora pansihalos, Otospora bareae, Palaeospora spainii, Paraglomus laccatum, Rhizoglomus invermaium, Rhizophagus intraradices, Rhizophagus irregularis, Septoglomus constrictum, S. nigrum, S. viscosum), or 28 species from 15 genera, as well as 24 virtual taxa at the species level.

The list of genera with the number of OTUs identified in the ITS1 region is presented in Table S5. The most common genera (54% OTUs) are highlighted in green; the group of minor genera is highlighted in blue (5% OTUs), which include *Ambispora*, *Otospora*, *Scutellospora*, *Palaeospora*, and *Halonatospora*.

The following known species were identified for ITS2 (Table S6):

- Subalpine meadow (Acaulospora alpina, Ac. brasiliensis, Ac. paulinae, Ambispora gerdemannii, Am. leptoticha, Archaeospora trappei, Claroideoglomus claroideum, C. lamellosum, Diversispora varaderana, Dominikia bernensis, Entrophospora infrequens, Glomus bareae, G. indicum, G. macrocarpum, Paraglomus laccatum, Rhizoglomus melanus, Rhizophagus intraradices, Rhizophagus invermaius, Rhizophagus irregularis), or 19 species from 11 genera, as well as 15 virtual taxa at the species level;
- Forest (Acaulospora paulinae, Ambispora gerdemannii, Am. leptoticha, Claroideoglomus claroideum, C. lamellosum, Diversispora slowinskensis, Entrophospora infrequens, Glomus hoi, G. indicum, Paraglomus laccatum, Rhizophagus intraradices, Rhizophagus irregularis, Scutellospora alterata), or 13 species from 9 genera, as well as 7 virtual taxa at the species level;
- River valley (Acaulospora paulinae, Ac. punctata, Ambispora fennica, Am. gerdemannii, Archaeospora spainiae, Ar. trappei, Cetraspora gilmorei, Claroideoglomus claroideum, Cl. hanlinii, Cl. lamellosum, Dominikia bernensis, Do. difficilevidera, Entrophospora infrequens, Funneliformis mosseae, Glomus hoi, G. indicum, G. macrocarpum, Halonatospora pansihalos, Paraglomus laccatum, Rhizophagus aggregatus, Rhizophagus intraradices, Rhizophagus invermaius, Rhizophagus irregularis, Scutellospora pellucida), or 24 species from 13 genera, as well as 20 virtual taxa at the species level.

The list of genera with the number of OTUs detected for region ITS2 is presented in Table S7. The most common genera (54% OTUs) are highlighted in green; the minor genera group (5% OTUs) is highlighted in blue. As shown in Tables S5 and S7, ~50% of all AM fungi OTUs are the OTUs belonging to only three genera: *Rhizophagus*, *Dominikia*, and *Glomus*, both in regions ITS1 and ITS2. The fungi from these genera were present in OTUs of meadows and river valleys but were absent in some cases in STPs belonging to forest biotopes. Minor genera (5% OTUs) for region ITS2 included *Scutellospora*, *Microkamienskia*, *Rhizoglomus*, *Diversispora*, *Cetraspora*, *Halonatospora*.

The construction of phylogenetic trees indicated that employment of ITS1 guaranteed the identification of more taxa at the species level. The reason is the specificity of primer annealing on the analyzed genetic markers. The sequence analysis revealed the presence of 560 OTUs identified up to the species level by regions ITS1 and ITS2 for nine STPs of three biotopes of the North Caucasus. However, 161 OTUs were excluded from the OTUs identified up to the species level due to the lack of clustering in phylogenetic trees constructed for individual AMF genera. While 285 OTUs were identified by up to 38 species from 16 genera and 27 virtual taxa at the species level via ITS1, 273 OTUs were identified by up to 32 species from 15 genera and 25 virtual taxa at the species level via ITS2. Therefore, the average number of detected OTUs for AMF in the sequences analysis of the ITS1 and ITS2 region was equal (slightly less for ITS2), while the total number of detected reads for the fungi of the Glomeromycetes class was 2.5 times higher via ITS1 than via ITS2 (Table 2).

According to the data of all the analyzed STPs, the following species were the most common in terms of calculation of reads for ITS1 + ITS2 regions: (1) *Dominikia bernensis* (>2000 reads); (2) *Glomus indicum*, *Rhizophagus intraradices*, and *Entrophospora infrequens* (>1000 reads for each species); (3) *Ambispora gerdemannii*, *Rhizophagus irregularis*, and *Paraglomus laccatum* (>500 reads for each species). Meanwhile, the calculation of the number of OTUs for the ITS1 + ITS2 regions indicates that the most common species were as follows: (1) *Rhizophagus intraradices* (51 OTUs); (2) *Paraglomus laccatum* (37 OTUs); (3) *Rhizophagus irregularis* (33 OTUs); (4) *Dominikia bernensis* (32 OTUs); (5) *Claroideoglomus claroideum* (21 OTUs); (6) *Funneliformis mosseae* (17 OTUs), and (7) *Acaulospora paulinae* (16 OTUs). The most common genera were (1) *Rhizophagus* and *Dominikia* (>3000 reads for >100 OTUs for each genus); (2) *Glomus* (>2000 reads for >50 OTUs); and (3) *Paraglomus* and *Claroideoglomus* (>500 reads for >30 OTUs). The list of endemic species is presented in Table S8. The results show that among the known species, 7 specific (endemic) species were found in the biotopes of the subalpine meadow, 4 in the forest, and 17 in the disturbed ecosystems and pastures in the biotopes of the river valley.

The Shannon and Simpson indices were used for a more accurate comparison of biodiversity in the studied biotopes (Table S9). The AMF species diversity indices calculated from the data obtained using two various markers differ from each other. All Hill numbers were higher for the data obtained for ITS1 than for ITS2. This was typical for both observed and extrapolated indices. The lowest observed diversity indices were noted for the forest ecosystem; the highest ones were for the river valley. The Hill indices (estimated by the sample size extrapolation) also revealed the low diversity in the forest ecosystem. The extrapolated value of species richness was maximal for the meadow. Still, the confidence interval value was also very high for the meadow, so it was complicated to conclude which of the two biotopes had a higher index. The extrapolated Shannon and Simpson indices were significantly (*p* < 0.01) higher for the river valley and significantly (*p* < 0.01) lower for the forest according to ITS2 data but close to each other in all three biotopes according to ITS1 data. This study also includes the comparison with both markers of the observed and extrapolated indices, which indicates that for the river valley the identification of more than 96% of the species diversity.

The results indicate that the analyzed Caucasus biotopes had different biodiversity and included a different number of specific species of AM fungi. Therefore, it is of interest to analyze the correlations between biodiversity for individual STPs and agrochemical parameters of sampled rhizospheric soils.

### 3.4. Correlation Analysis of the Interlinks between AMF Species Richness with STP Height and Agrochemical Parameters of Sampled Rhizosphere Soils

The assumption of this study was the existence of a correlation of species richness with soil agrochemical indicators and STP height (altitude). The analysis was performed with application of *rho*, Spearman correlation (Figures S10 and S11). The results indicate the existence of a direct positive correlation of AMF biodiversity with the pH value only. The highest greatest correlation was found for the OTUs in the ITS1 region (Figure S10). We did not find any correlation between other soil parameters and the altitude above sea level. Linear correlation coefficients (*r*) between the AMF biodiversity parameters, plant biodiversity, and agrochemical indicators of STPs are presented in Table 3. Proven (*p* < 0.05) positive correlations were found between the indicators of AMF diversity with “Percentage of annual plants”, with “pHKCl”, and in some cases with “Pi, mg/kg” (inorganic phosphorus available for plant nutrition).

## 4. Discussion

### 4.1. Applicability of Molecular Genetic Identification of AMF in the Study of Their Biodiversity

Our study focuses on defining the AMF biodiversity in the North Caucasus, a temperate biodiversity hotspot for plants and animals, to detect new AMF hotspots. The correct identification of individual AMF species is possible via the morphological method. However, AMF do not grow on culture media, so the accuracy of estimating the AMF species richness by this method is rather low. In this regard, we carried out the identification using molecular genetic methods with Illumina MiSeq. The results reveal that the significant differences among the three analyzed biotopes for the Glomeromycetes class include all known AMF species. A sustainability assessment and approach relevance for molecular genetic identification of AMF, its advantages, and disadvantages will be discussed further. We propose a new methodology to solve the issue of high AM fungi polymorphism for ITS regions by constructing trees by genus, accompanied with manually verified alignment (Figure S1). It is impossible to construct a phylogenetic tree for the whole Glomeromycetes class for ITS regions, so the AM fungi studies mostly took into account the conservative genes, for example, the LSU region [81]. The LSU region is also only partially suitable for constructing a complete reference tree for Glomeromycetes. In the investigation of Delavaux et al., this tree included only 174 sequences of AM fungi assigned to only 112 species [81], even though 345 species of AMF are known [32]. Thus, this reference tree is insufficient to identify most species (more than ⅔ of known AMF species). In this case, the assessment of AMF biodiversity and their identification only via LSU at the species level is not yet possible, and most studies are currently associated with the definition of AMF taxa to the genus level [16,19,24,81]. The SSU region is believed to be slowly evolving, and therefore not sufficiently variable to adequately identify AMF species [82,83,84]. As suggested in this study, the algorithm (Figure S1) eliminates the disadvantage of using polymorphic ITS regions to carry out correct clustering for AMF identification at the species level. Currently, ITS1 and ITS2 are, as a rule, analyzed unequally [33]. Thus, in the NCBI GenBank database, individual sequences of the ITS2 region are the most frequent, and sequences of the ITS1 region are represented as part of the entire SSU-ITS1-5.8-ITS2-LSU region. According to the data of our study, the ITS1 analysis demonstrated a significant number of reads, and it can be assumed as a second barcoding region along with the ITS2 region. It is highly probable that the process of primer annealing for the ITS1 and ITS2 regions varies, as indicated by the distinct compositions of the identified AMF species (Tables S4 and S6). It is assumed that analyzing the ITS1 and ITS2 regions would complement each other as if they were distinct genetic markers.

In the biotopes of the North Caucasus, for ITS1 and ITS2, the total number of AMF species was 50 (the number of clades of the species level with accurate identification) from 20 genera. Currently, we know of 345 AMF species from 44 genera [32]. Thus, at least 15% of the world’s AMF species diversity has been identified in the studied region of the North Caucasus (from 45% of genera). AM has been extensively studied for several decades, including in the North Caucasus and other regions of Russia. The Teberdinsky National Park plant communities, including arbuscular mycorrhizae, have been studied for many years by Onipchenko et al. [85,86,87]. Authors discovered that about 74–77% of the higher plants of the National Park interact with AMF, forming a symbiosis, arbuscular mycorrhiza [87]. However, a detailed assessment of the diversity of AMF species in the North Caucasus has not yet been performed. Evaluating biodiversity and comparing different floral complexes (river valley, mountain forest, and subalpine meadow) was difficult without applying molecular genetic methods. The main approaches were at the level of classical floristics, geobotany, and mycology, known as methods of comparative morphological analysis based on species identification only by the morphology of rhizospheric extraradical AMF spores, but not by intraradical AM structures: vesicles, arbuscules, and mycelium [88]. Meanwhile, the AMF peculiarities concern their obligate status in relation to the host plant and the fundamental impossibility of growing AMF spores in soils without plants. Therefore, it is very difficult to assess AMF biodiversity by the morphology of spores. On the other hand, the difficulties of molecular genetic identification of AMF by rDNA regions are linked to the fact that the concepts of “organism” or “species” are not fully applicable to AMF [40] because AMF contain nuclei of different origins in their hyphae and spores (heterokaryosis), i.e., essentially one fungus contains a set of polymorphic genotypes, and since the diversity of rDNA evaluates the analysis of genetic diversity, it more correct a definition that one AM fungus have a set of different ribotypes. Considering the community of nuclei within the arbuscular mycorrhiza in the studied STP, it is appropriate as a peculiar pan-genome, rapidly changing due to horizontal gene transfer [89,90,91]. It is also known that AMF can form anastomoses [92] and, accordingly, the exchange of genetic material. All this is the reason that different OTUs belong to the same AMF species according to the sequencing results obtained in this and earlier studies [63]. Thus, molecular genetic methods, including NGS sequencing using Illumina MiSeq, have advantages in assessing AMF biodiversity up to the species level in comparison with morphological methods of AMF identification (Table S10; [33,93,94]). Nevertheless, only morphological methods provide reliable information for accurate species identification. Some disadvantages of NGS sequencing should also be mentioned (Table S10). The main problem is the high genetic polymorphism of AM fungi.

The genetic mechanisms of heterogeneous nuclei interaction within a single pan-genome have not been studied. Three different phenomena may cause the OTU genetic diversity. Firstly, it is a consequence of the heterogeneity of nuclei in hyphae and AMF spores. For example, one spore can contain up to 35,000 nuclei [95]. The reason is that AM fungi (unlike other eukaryotes) lack the genetic bottleneck of a single-nucleus stage [96].

Secondly, the detected heterogeneity of the AMF genetic material may result from the genome conflict, a “genomic shock” (see the studies on hybrids and plant allopolyploids). At the rDNA level, it is expressed as “nucleolar dominance”, known as the silencing of the rDNA of one of the parents, a high rate of accumulation of substitutions in the repressed genome, and a gradual decrease in the proportion of rDNA of the repressed subgenome in the polyploid genome [97,98,99]. Such a phenomenon of nucleoli dominance is also found in AM fungi [100]. It is can be seen by the appearance of a high number of singletons—unique and quasi-unique rDNA variants, represented among reads by a minimum number of copies [97], similar to what was found in this study. The provided identification of a larger number of OTUs for ITS1 is due to the greater ITS1 variability, and, secondly, since AMF contain many nuclei that have different variants of both ITS1 and ITS2. Consequently, the same mycelium of the same AMF species may contain more variants of ITS1 than ITS2. The different variability of the regions (the rate of changes in ITS2 is less than in ITS1) and the different specificity of primers indicate that the complex analysis of both regions significantly complements each other. Singletons often contain chimeric sequences, but their exclusion can lead to a significant loss of truly existing fungi species. This was estimated in the analysis by Baldrian et al. since 2.5% of 10 million singletons had a more than 97% similarity to fungi species [27].

The third mechanism that can lead to the increase in the diversity of OTUs in the studied STPs (in addition to the real taxonomic diversity of the mycota of each site) is a parasexual process that naturally directs to the exchange of genes between nuclei during karyokinesis [101]. The common opinion was that mycorrhizal fungi are asexual. However, the preservation in their genomes of 85% of gene complexes associated with crossing over of sexual forms [102] fully admits the assumption that a hidden parasexual process is possible [88,103].

### 4.2. Comparative Analysis of AMF Diversity in different biotopes

**Figure 2:**
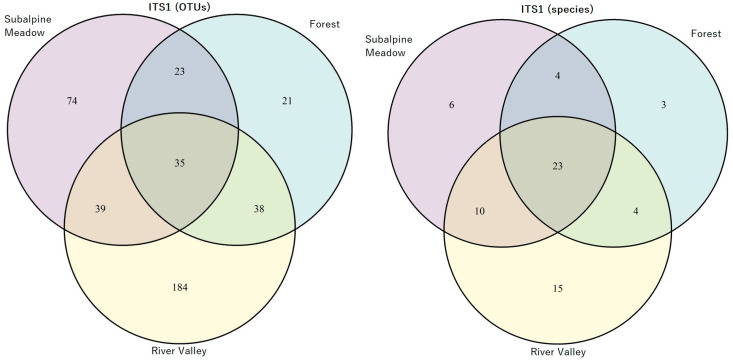
Venn diagram: the number of identified OTUs (**left**) and AMF species (**right**) in subalpine meadow, forest, and river valley based on ITS1 analysis.

**Figure 3:**
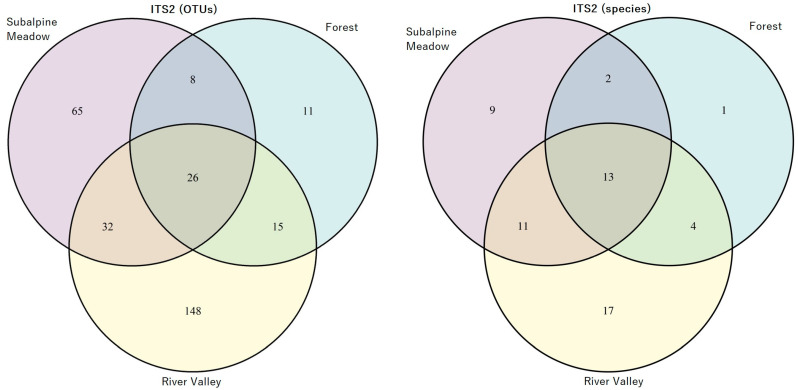
Venn diagram: the number of identified OTUs (**left**) and AMF species (**right**) in subalpine meadow, forest, and river valley based on ITS2 analysis.

As a result of the OTUs and phylogenetic tree analysis, the AMF species were identified (Tables S4 and S6). The OTUs and AMF species’ relative abundance in three biotopes (subalpine meadow, forest, and river valley) is represented in Figure 2 and Figure 3 for the ITS1 and ITS2 regions, respectively. The most significant number of specific AMF species was found in the river valley, with the minimum in forest biotopes for both ITS regions, including the calculation of particular species without VT (Table S8). The highest number of species was identified in all biotopes for the ITS1 region compared to ITS2.

Among the biotopes of meadows, forests, and river valleys, both endemic and common AMF species for different biotopes were identified (Table S8). The dominant AMF species in the North Caucasus ecosystems are (in descending order of the number of OTUs, species with the number of identified OTUs > 350): *R. intraradices*, *R. irregularis*, *Entrophospora infrequens*, *Dominikia indica*. Whereas the conclusions about the taxon distribution based on the number of OTUs are risky, they may be correct for dominant and subdominant species. For example, such AMF genera as *Rhizophagus* and *Dominikia* are recognized as most common (by the number of OTUs and reads), so they should be considered cosmopolitan genera. According to data from the literature [104], *R. irregularis* and *R. intraradices* species have been identified in many ecosystems of the world. The *Entrophospora* genus should be mentioned separately. This is a monophyletic group that has recently been included in AMF [105] and assigned to the new order of *Entrophosporales* (according to several sources, along with *Claroideoglomus*; [41]). The study of the *Paraglomus* genus is of a particular interest because its sequences in the NCBI database for ITS regions are not enough for accurate species identification (there are only three species out of nine known by the morphology of spores in the database). All AMF of these genera have a significant number of reads in the data sets obtained for the STPs of the North Caucasus (Tables S4 and S6). Furthermore, the list of the most represented genera in the ecosystems of the Andes [58] differs from genera in the biotopes of the North Caucasus (*Rhizophagus*, *Dominikia*, and *Glomus*; see Tables S5 and S7). The following genera can be found in the Andes: *Acaulospora* (>50% reads; the genus is characteristic of high zones), *Claroideoglomus*, *Cetraspora*, and *Rhizophagus* [58]. In the Southern Central zone of Chile, the main AMF genera were *Acaulospora* and *Glomus* [43]. In Yucatan, Mexico, the main AMF genera associated with tree roots were *Glomus*, *Sclerocystis*, *Rhizophagus*, *Redeckera*, and *Diversispora* [19]. In Mt. Taibai of Qinling Mountain, China, the main AMF genera were *Glomus*, *Septoglomus*, *Acaulospora*, *Ambispora*, and *Rhizophagus* [106]. We can assume that mountain biotopes differ significantly in the composition of AMF genera.

A comparative analysis of biotopes revealed common features in the species composition for ecosystems of the same type (Table S4 and S6). In addition to the known species, we identified 52 species-level VTs assigned to 12 genera (for the ITS1 and ITS2 regions). Virtual taxa found in the ecosystems of the North Caucasus, a temperate biodiversity hotspot, are also important because they may represent new, previously unknown, uncharacterized AMF species. So, in the study carried out in Yucatan, Mexico, Lara-Pérez et al. [19] identified 36 VTs, belonging to nine genera (for 18S region). In the Serengeti National Park, Tanzania, Stevens et al. [23] identified 39 VTs (for the SSU region); in the High Arctic, the Zackenberg Valley, Northeast Greenland, Rasmussen et al. [44] identified 29 VTs (for the SSU region); in the Middle Caquetá River region and the municipality of Leticia, the Colombian Amazon region, Peña-Venegas et al. [22] identified 126 VTs (for SSU region). Still, the application of SSU as a barcode did not allow for the identification of AMF to the species level. The composition of morphological and molecular genetic approaches can significantly expand the possibilities of AMF identification. For example, Vieira et al. [29] found as many as 62 AMF species from 18 genera (for the 25S region) in the Iron Triangle region, Brazil.

According to experimental results, for any data calculation (reads, OTUs, the number of precisely defined AMF species, the total number of species taking into account the number of virtual AMF taxa at the species level, the values of biodiversity indices), forest ecosystems have the least AMF biodiversity, and river valley ecosystems, on the contrary, have the highest AMF biodiversity. However, according to the estimated species richness (Table S9), the subalpine meadow had equally high diversity with that of river valley meadows. Based on the analysis of over 2 million valid sequences with an average sequence length of 216 bp for each sample, it was shown that the subalpine meadow (2635 m above sea level) on the western slope of Helan Mountain in Alxa Left Banner, Inner Mongolia Autonomous Region, had the highest diversity according to the Shannon index and the ACE and Chao1 indices. However, it manifested the smallest Simpson index [50].

### 4.3. Reasons for Higher AMF Diversity in River Valley Biotopes

To elucidate the reasons for the taxonomic diversity of AMF, we analyzed several factors that could be important. Such abiotic and biotic factors include climatic conditions, agrochemical composition of soils, human activity, and composition of plant communities [47,48,49,50]. The provided multivariate analysis of variance revealed that the number of OTUs in the space clusters the STPs of one biotope (subalpine meadow/forest/river valley) obtained using multidimensional scaling (MDS-Spearman). Correlation analysis indicated a significant (*p* < 0.01 for ITS1) positive relationship between soil acidity (pH_KCl_) and AMF species richness (Figures S10 and S11) and in some cases of AMF, biodiversity indicators with the level of available *p* for plants (“Pi, mg/kg”; Table 3). The effect of other agrochemical soil parameters and the altitude of the STPs was weaker or absent (Table 3).

The list of possible reasons for high AMF diversity in a river valley:

- Livestock grazing and other human activities are more prevalent in river valleys compared to mountain forests and subalpine meadows [14]. Due to this, it is common to see the transfer of spores of AM fungi through human shoes and livestock hooves (such as cows and horses) [49,107]. However, some evidence indicates that ungulate grazing may be associated with decreased AM fungal abundance in soil [23]. Additionally, AMF spores can migrate with water flows from the mountains to the river valley during erosions [107,108,109], and they actively enter into nonspecific symbiotic relationships after sedimentation. Despite the fact that the organic reserves in these ecosystems are much higher because of livestock grazing, the ecosystems themselves may have signs of soil degradation and are considered disturbed. Nevertheless, AM fungi spores, as a rule, are significantly larger (>40 microns) than the spores of many other fungi, so their distribution distance is relatively short [110]. According to Guo et al. [111], terrain slope can also affect AMF diversity. The biotopes of the river valley analyzed in our study were characterized by a much gentler slope than the biotopes of subalpine meadows and forests (Table S2). Therefore, it can be assumed that a flat slope will positively correlate with AMF biodiversity.
- In conditions of intensive percolation water regime and good drainage, there is no stagnation of water and oxygen deficiency in the soil, negatively affecting the development of AM fungi [112,113]. The percolation water regime can reduce the content of available phosphate (Pi) in the soil, which makes root mycorrhization an important adaptation for P uptake. Such a water regime is typical for river valleys in the North Caucasus. The correlation between the seven parameters of AMF biodiversity and “Pi, mg/kg” (inorganic P available for plant nutrition) in many cases was significantly (*p* < 0.05) positive (Table 3). Our data are consistent with the results of Guo et al. who found that the diversity of AMF had a positive correlation with available P content [111]. Nevertheless, large-scale studies have shown that AMF diversity and abundance decrease with the phosphorus available in the soil [114]. This issue requires further consideration.
- STP altitude may be a factor that influences AM development. A reduced number of identified AMF at the species level (Figure 2 and Figure 3) characterized the analyzed biotopes of the forest and subalpine meadow located above the biotopes of the river valley. However, linear reliable correlations for the North Caucasus STPs were not found (Table 3). Our results are consistent with the available data [12,115,116,117,118]. AMF biodiversity was largely unaffected by altitude [51,55,56,57,58], with some negative correlations found [12,115,116,117,118]. However, this rule is not applicable for the Zackenberg Valley in the High Arctic [44]—with an increase in altitude from 33 to 479 m (a small altitude above sea level), an increase in AM fungi occurrence was observed. Perhaps the reason for the lack of correlation between altitude and AMF biodiversity in the North Caucasus is that there are no biotopes of alpine meadows in the analyzed STPs, which are characterized by lower temperatures. However, temperature is considered an important factor for the development of AM [113,119,120]. The optimal average air temperature of the warmest month is +19 °C, but mycorrhizal colonization can be intensively increased with the frosty period not less than 2 months [121].
- The biodiversity of AMF and the development of AM are affected by such factors as pollution, salinity, drought, extreme temperatures, CO_2_, liming, acidity, etc. [122], as well as soil composition, altitude, species composition of plant communities, and climatic factors [47,48,50]. However, pH and temperature are the main factors determining AMF biodiversity [60]. pH can have an important direct effect on the growth and productivity of the AM fungus [123,124]. The positive correlation between AMF diversity and pH is mentioned in [111,125]. This is consistent with our data on a significant positive correlation of the pH_KCl_ with a number of biodiversity indicators (“Glomeromycota total reads”, “OTUs number for total species”, “total species number”, both for ITS1 and ITS2; Table 3; Figures S10 and S11).
- The phenotypic diversity of OTU AMF is supposed to be under the effect of the phenotypic diversity of plants, which decreases with altitude in the mountains [126]. The colonization of roots by AM fungi is not species–specific. Several AM fungi species can colonize one plant’s root, and one AM fungus can colonize different plant species [127]. However, despite the absence of direct correlations between the diversity of AMF and the total number of herbaceous plants (Table 3), a decrease in the spectrum of potential partners in the mutualistic symbiotic system might be expected, which affects the AMF species diversity [128]. At the same time, annual plant species have a higher diversity of AMF than perennial species, and a half of the currently identified AMF species may be more specific to one plant species [54]. Moreover, S. Horn et al. [51] demonstrated that the influence of the biotic factors (interaction of AMF with plants) is more significant in comparison with the effect of abiotic factors on the AMF genera composition. It is known that in the process of succession with an increase in the proportion of woody plants, the density of AM fungi spores decreased [52,53]. Thus, higher abundance of annual plants (see “Percentage of annual plants” in Table S2) in river valleys in comparison with the biotopes of the subalpine meadow may be a key factor positively affecting AMF taxonomic diversity. Meanwhile, our studies confirm the relationship between the proportion of annual plant forms and the diversity of AMF. For instance, it was shown that the linear correlation coefficients were reliable (*p* < 0.05; Table 3). The correlation coefficient between “Percentage of annual plants” and “Glomeromycota total reads” *r* = 0.76 and 0.81 (for ITS1 and ITS2, respectively), and the correlation coefficient between “Percentage of annual plants” and “OTUs number (for total species)” *r* = 0.67 and 0.77 (for ITS1 and ITS2, respectively). A weak correlation between the proportion of annual plants and the AMF species diversity was also shown (*r* = 0.40 and 0.56 for ITS1 and ITS2, respectively; see Table 3). Similar results were obtained in [43]; the “natural grassland” ecosystem had the highest AMF species diversity among 20 ecosystems of interest. The opposite is also possible to occur. It is shown that in the Teberdinsky National Park, experimental suppression of AM symbiosis is always followed by a decrease in the species richness and number of plants [129]. The species composition and numerical relative abundances of different OTUs may vary due to seasonal changes, so observing OTU diversity in different seasons can provide new information [130,131]. Changes in the mycorrhization of plants by AM fungi throughout a year in the Teberdinsky National Park were already studied earlier [85], but their biodiversity has not been assessed.

### 4.4. Practical Application of the Results of AMF Biodiversity Research

*Rhizophagus irregularis*, *Funneliformis mosseae*, and *Gigaspora margarita* are the species most often used in biological preparations to enhance plant growth [45,132,133,134,135,136]. Meanwhile, the results of this study show that among 10 million sequences, the ones with *F. mosseae* and *Gi. margarita* are extremely rare, requiring the special attention of researchers because these fungi are often isolated from soils by morphological methods. By isolating AMF from the analyzed STPs, we collected a large number of *R. irregularis* spores, a few *F. mosseae* spores, but failed to isolate *Gi. margarita* spores from the roots of plants in the North Caucasus (unpublished data). Universal primers are, perhaps, less suitable for sequencing these two species. Other authors reported that these species may be absent [24,25] or rarely occur in the sequencing results, even if they are identified by morphological methods [11,19,23], or not all species of *Gigaspora* and *Funneliformis* genera are found [18,22,29]. Thus, we can assume that the application of universal primers (for ITS1 and ITS2 regions) makes assessing AMF biodiversity possible. When studying a single new species, it may be useful to analyze, for example, the LSU region [81] as another barcoding region.

## 5. Conclusions

The investigation of the North Caucasus region, in the Teberdinsky National Park and adjacent territories of Karachay-Cherkessia, revealed a significant number of AMF species were identified for the first time: 50 species from 20 genera in three biotopes, subalpine meadow, forest, and river valley. This represents at least 15% of the world species diversity of AMF (from 45% of known genera). Thus, the biotopes of the North Caucasus should be considered as a native temperate biodiversity hotspot not only for plants and animals but also for AM fungi. Modification of the molecular genetic identification of AMF showed the effectiveness of the proposed algorithm (Figure S1), including (1) using Illumina MiSeq simultaneously for two barcodes (ITS1 and ITS2 regions); (2) the inclusion of analysis for genera phylogenetic trees as a separate step as a part of species identification algorithm; (3) the selection of sequences from the MaarjAM database as OTU centroids, which made it possible to use about 15% of reliable singletons and thereby minimize losses of true AMF species. The list of species identified in all biotopes includes *Acaulospora paulinae*, *Claroideoglomus claroideum*, *C. lamellosum*, *Dominikia indica*, *Glomus hoi*, *Paraglomus laccatum*, *Rhizophagus irregularis*, *R. melanus*, and *R. intraradices*. The dominant species, *R. intraradices*, *R. irregularis*, and *D. indica*, and the specific (endemic) species were identified: 7 species for biotopes of the subalpine meadow, 4 in the forest, and 17 for disturbed ecosystems, pastures in the river valley biotopes (Table S8). In addition, 52 virtual taxa at the species level were also found and assigned to 12 genera. These taxa may include some previously unknown AMF species. A correlation analysis revealed that the main reasons for high biodiversity in river valleys can be as follows: (1) a higher proportion of annual plant species in river valleys in comparison with the biotopes of subalpine meadows and forests, since it is annual plant species that have a higher diversity of AMF than perennial ones; (2) the disturbance of river valley biotopes (active grazing on pastures) in contrast to undisturbed biotopes of forests and subalpine meadows, which is a sign of the process of regenerative succession, in which the role of AMF is high; (3) the positive correlation of AMF diversity with pH in the soil. Our data prove the importance of future investigations in the North Caucasus region, in the Teberdinsky National Park, and the adjacent territories of Karachay-Cherkessia. The accumulated data will expand our knowledge about the role of AMF involvement in the formation of natural biotopes.

## Figures and Tables

**Table 1:** Locations and soils for analyzed Stationary Trial Plots.

STP Number	Stationary Trial Plot	Coordinates	Altitude, m	Type of Soil	Soil Profile
1	Subalpine Meadow-4, Malaya Hatipara ridge	43°25′50.0″ N 41°42′20.0″ E	2437	ID 199 Mountain-meadow sod-peaty WRB, 2006. Umbric Leptosols FAO, 1988. Umbric Leptosols	O1/A1v-A1Bp-BCp-Cp
3	Subalpine Meadow-3, Malaya Hatipara ridge	43°25′48.0″ N 41°42′31.0″ E	2401	ID 200 Mountain-meadow soddy WRB, 2006. Umbric Leptosols FAO, 1988. Umbric Leptosols	A1-A2-B
4	Subalpine Meadow-2, Malaya Hatipara ridge	43°25′51.0″ N 41°42′55.0″ E	2186	–//–	–//–
7	Fir Forest-3, Malaya Hatipara mountain	43°26′07.3″ N 41°43′14.1″ E	1900	ID 68 Brownzems raw-humic illuvial-humic WRB, 2006. Haplic Cambisols FAO, 1988. Dystric Cambisols	O(AO)-A1-A1A2-Bm,f,h(Bh,m)-C
8	Pine Forest-3, Malaya Hatipara mountain	43°26′07.3″ N 41°43′14.1″ E	1890	–//–	–//–
9	Mixed forest near the Bolshaya Hatipara river, Bolshaya Hatipara mountain	43°24′56.0″ N 41°42′49.0″ E	1507	–//–	–//–
11	Grassland in the valley of the Teberda river, Teberda town	43°25′12.0″ N 41°43′45.0″ E	1342	ID 191 Alluvials compact WRB, 2006. Gleyic Vertisols FAO, 1988. Eutric Vertisols	A1v-A1-Bve-BC-C
12	Grassland in the valley of the Teberda river, the border of the New Teberda village	43°39′37.0″ N 41°53′12.0″ E	1026	ID 188 Alluvials saturated WRB, 2006. Haplic Fluvisols FAO, 1988. Eutric Fluvisols	A1-AB-B-BC-D
13	Grassland in the valley of the Kuban river, Ordzhonikidzevsky village	43°51′38.0″ N 41°54′22.0″ E	795	–//–	–//–

Note: “–//–”—the same as above.

**Table 2:** The number of fungal reads and OTUs for ITS1 and ITS2 in the analyzed samples of 9 STPs.

Analyzed	Subalpine Meadow			Forest			River Valley			Total for 9 STPs
Parameters	STP 1	STP 3	STP 4	STP 7	STP 8	STP 9	STP 11	STP 12	STP 13	
Total reads	1,494,700	619,855	1,648,642	652,998	1,469,158	970,340	1,108,444	1,271,980	862,545	10,098,662
Merged reads after length trim	401,850	255,057	626,988	102,472	586,935	539,334	363,547	160,449	347,435	3,384,067
Glomeromycota ITS1 total reads	1243	1011	1116	183	76	697	6670	335	4638	15,969
Glomeromycota ITS1 OTU number	100	59	86	29	26	80	197	66	109	414
	171			117			296			
Glomeromycota ITS2 total reads	546	493	580	7	24	583	1624	885	1758	6500
Glomeromycota ITS2 OTU number	72	49	74	3	10	58	128	93	76	305
	131			60			221			

Note: The values of the number of OTUs before filtering the sequences unidentified at the species level are presented. The significance of dissimilarity in ITS compositions (the OTU number) analyzed for different biotopes, assessed as Spearman’s distances (1-r), was confirmed via ANOSIM tests (*p* = 0.003 for ITS1 and *p* = 0.004 for ITS2). After identifying AMF to the species level, the out statistics are provided in Tables S4–S7.

**Table 3:** Linear correlation coefficients (*r*) between AMF biodiversity parameters, plant biodiversity parameters, and agrochemical indicators of STPs (“*”—significant value, *p* < 0.05).

Analyzed Parameter	Parameter Number	1	2		3	4		5	6		7	8	9		10	11
The number of herbaceous plant species	1	1														
Percent of annual plants	2	−0.20	1													
Total reads	3	0.08	−0.09		1											
Merged reads after length trim	4	−0.10	−0.30		0.64	1										
Altitude, m	5	0.17	−0.76 *		0.18	0.21		1								
pHKCl	6	0.49	0.68 *		0.09	−0.17		−0.41	1							
Pi, mg/kg	7	−0.31	0.61		−0.04	0.05		−0.40	0.38		1					
Ntotal, %	8	0.21	−0.67 *		0.11	0.46		0.78 *	−0.31		−0.13	1				
Ptotal, %	9	0.23	−0.14		−0.19	0.37		0.29	0.18		0.36	0.72 *	1			
Sum of fractions < 0.01 mm	10	0.53	−0.31		0.40	−0.04		0.53	0.00		−0.53	0.31	−0.13		1	
Soil type by mechanical composition	11	−0.09	0.56		0.14	0.07		−0.88 *	0.31		0.47	−0.61	−0.25		−0.43	1
Glomeromycota ITS1 total reads	12	−0.15	0.76 *		−0.13	−0.01		−0.47	0.69 *		0.72 *	−0.34	0.31		−0.57	0.31
Glomeromycota ITS1 total OTU number	13	−0.01	0.64		0.10	0.10		−0.32	0.74 *		0.80 *	−0.11	0.42		−0.44	0.29
OTUs number for ITS1 (for total species)	14	−0.10	0.67 *		0.09	0.13		−0.33	0.71 *		0.81 *	−0.12	0.42		−0.47	0.29
OTUs number for ITS1 (species level without VT)	15	−0.17	0.68 *		0.02	0.11		−0.36	0.65		0.87 *	−0.11	0.45		−0.52	0.32
OTUs number for ITS1 (only VT species level)	16	0.18	0.50		0.34	0.20		−0.17	0.80 *		0.47	−0.15	0.25		−0.21	0.16
Species number for ITS1 (total)	17	0.07	0.40		0.41	0.38		−0.06	0.67 *		0.64	0.10	0.45		−0.23	0.15
Species number for ITS1 (without VT)	18	−0.03	0.40		0.31	0.42		−0.12	0.60		0.68 *	0.15	0.54		−0.37	0.18
Species number for ITS1 (only VT)	19	0.18	0.38		0.50	0.32		0.01	0.69 *		0.57	0.04	0.33		−0.06	0.11
Glomeromycota ITS2 total reads	20	0.13	0.81 *		−0.09	−0.07		−0.68 *	0.87 *		0.57	−0.44	0.22		−0.45	0.52
Glomeromycota ITS2 total OTU number	21	0.34	0.62		0.23	0.03		−0.40	0.89 *		0.67 *	−0.17	0.30		−0.15	0.46
OTUs number for ITS2 (for total species)	22	0.20	0.77 *		0.04	−0.02		−0.60	0.92 *		0.53	−0.37	0.22		−0.34	0.47
OTUs number for ITS2 (species level without VT)	23	0.05	0.78 *		−0.10	−0.00		−0.64	0.82 *		0.54	−0.39	0.26		−0.49	0.45
OTUs number for ITS2 (only VT species level)	24	0.57	0.45		0.44	−0.06		−0.26	0.85 *		0.30	−0.19	0.02		0.24	0.35
Species number for ITS2 (total)	25	0.54	0.51		0.26	0.02		−0.36	0.91 *		0.40	−0.10	0.27		0.03	0.39
Species number for ITS2 (without VT)	26	0.41	0.56		0.08	0.04		−0.41	0.87 *		0.45	−0.02	0.44		−0.11	0.36
Species number for ITS2 (only VT)	27	0.62	0.37		0.46	−0.02		−0.24	0.82 *		0.26	−0.19	0.01		0.21	0.37
**Analyzed Parameter**	**Parameter Number**	**12**	**13**	**14**	**15**	**16**	**17**	**18**	**19**	**20**	**21**	**22**	**23**	**24**	**25**	**26**
The number of herbaceous plant species	1															
Percent of annual plants	2															
Total reads	3															
Merged reads after length trim	4															
Altitude, m	5															
pHKCl	6															
Pi, mg/kg	7															
Ntotal, %	8															
Ptotal, %	9															
Sum of fractions < 0.01 mm	10															
Soil type by mechanical composition	11															
Glomeromycota ITS1 total reads	12	1														
Glomeromycota ITS1 total OTU number	13	0.90 *	1													
OTUs number for ITS1 (for total species)	14	0.93 *	0.99 *	1												
OTUs number for ITS1 (species level without VT)	15	0.92 *	0.98 *	0.99 *	1											
OTUs number for ITS1 (only VT species level)	16	0.80 *	0.88 *	0.87 *	0.79 *	1										
Species number for ITS1 (total)	17	0.75 *	0.92 *	0.91 *	0.87 *	0.93 *	1									
Species number for ITS1 (without VT)	18	0.77 *	0.93 *	0.93 *	0.90 *	0.87 *	0.98 *	1								
Species number for ITS1 (only VT)	19	0.68 *	0.86 *	0.84 *	0.78 *	0.93 *	0.97 *	0.89 *	1							
Glomeromycota ITS2 total reads	20	0.88 *	0.82 *	0.82 *	0.80 *	0.77 *	0.65	0.67 *	0.59	1						
Glomeromycota ITS2 total OTU number	21	0.70 *	0.88 *	0.84 *	0.81 *	0.82 *	0.82 *	0.79 *	0.82 *	0.82 *	1					
OTUs number for ITS2 (for total species)	22	0.85 *	0.85 *	0.84 *	0.80 *	0.84 *	0.73 *	0.74 *	0.68 *	0.98 *	0.88 *	1				
OTUs number for ITS2 (species level without VT)	23	0.90 *	0.82 *	0.83 *	0.81 *	0.78 *	0.67 *	0.71 *	0.58	0.99 *	0.76 *	0.97 *	1			
OTUs number for ITS2 (only VT species level)	24	0.36	0.60	0.53	0.47	0.69 *	0.63	0.53	0.71 *	0.59	0.88 *	0.70 *	0.51	1		
Species number for ITS2 (total)	25	0.53	0.72 *	0.67 *	0.62	0.75 *	0.71 *	0.67 *	0.71 *	0.77 *	0.94 *	0.85 *	0.71 *	0.93 *	1	
Species number for ITS2 (without VT)	26	0.61	0.76 *	0.72 *	0.69 *	0.70 *	0.69 *	0.71 *	0.62	0.83 *	0.89 *	0.89 *	0.81 *	0.78 *	0.95 *	1
Species number for ITS2 (only VT)	27	0.34	0.57	0.50	0.42	0.69 *	0.62	0.51	0.71 *	0.57	0.86 *	0.67 *	0.48	0.99 *	0.91 *	0.73 *

## Data Availability

Data are contained within the article.

## Supplementary Materials

The following are available online at https://www.mdpi.com/article/10.3390/jof10010011/s1, Figure S1: The algorithm of AM fungi identification; Table S1: Soil characteristics for analyzed Stationary Trial Plots in the sampling horizon 0–10 cm; Table S2: Characteristics of Stationary Trial Plots and plant communities; Table S3: Average and largest *p*-distances in ITS1 and ITS2 regions for various AMF genera; Figure S2. Samples representation in low dimensional space revealed from MDS (Multidimesional scaling). Spearman’s distance (1-rho) was used as metric of dissimilarity between OTUs profiles (A–ITS1, B–ITS2); Figure S3. Score plots from PCA of OTUs profiles (A–ITS1, B–ITS2); Figure S4. The relative abundance (%) of OTUs of main fungal phyla in regions ITS1 (A) and ITS2 (B) in three tested biotopes; Figure S5. The relative abundance (%) of OTUs of various fungal classes in regions ITS1 (A) and ITS2 (B) in three tested biotopes; Figure S6: Maximum Likelihood phylogenetic tree that represents OTUs of *Ambispora* genus identified in ITS1 region; Figure S7: ML phylogenetic tree that represents OTUs *Ambispora* genus identified in ITS2 region; Figure S8: ML phylogenetic tree that represents OTUs of *Acaulospora* genus identified in ITS1 region; Figure S9: ML phylogenetic tree that represents OTUs of *Acaulospora* genus identified in ITS2 region; Figure S10: Dependencies between agrochemical parameters of rhizosphere soil and numbers of species and OTUs (for ITS1 region); Figure S11: Dependencies between agrochemical parameters of rhizosphere soil and numbers of species and OTUs (for ITS2 region); Table S4: Identification of arbuscular mycorrhizal fungi: species list according to OTUs founded with ITS1 analysis; Table S5: Identification of arbuscular mycorrhizal fungi: genera list according to OTUs founded by ITS1 analysis; Table S6: Identification of arbuscular mycorrhizal fungi: species list according to OTUs founded with ITS2 analysis; Table S7: Identification of arbuscular mycorrhizal fungi: genera list according to OTUs founded by ITS2 analysis; Table S8: List of common and endemic species (except VT) ranked in decreasing order of OTUs; Table S9: Observed and extrapolated values of species richness, Shannon diversity, and Simpson diversity by OTUs; Table S10. Advantages and disadvantages of molecular genetic methods using Illumina MiSeq to identify AMF species in comparison with morphological methods.
