# Supplementary material for: Diversity of Arbuscular Mycorrhizal Fungi in Distinct Ecosystems of the North Caucasus, a Temperate Biodiversity Hotspot
Source: J Fungi (Basel). 2023 Dec 24;10(1):11. doi: 10.3390/jof10010011 (PMC10817546; doi:10.3390/jof10010011)
Supplement: Supplementary file 1 [file jof-10-00011-s001.zip › jof-2744710-supplementary.pdf]

Journal of Fungi

Supplementary Materials: The following are available online at https://www.mdpi.com/article/10.3390/jof10010011/s1-s17,

Figure S1: The algorithm of AM fungi identification,

Table S1: Soil characteristics for analyzed stationary trial plots in the sampling horizon 0-10 cm,

Table S2: Characteristics of stationary trial plots and plant communities,

Table S3: Average and largest *p*-distances in ITS1 and ITS2 regions for various AMF genera,

Figure S2. Samples representation in low dimensional space revealed from MDS (Multidimesional scaling). Spearman's distance (1-rho) was used as metric of dissimilarity between OTUs profiles (A – ITS1, B - ITS2).

Figure S3. Score plots from PCA of OTUs profiles (A – ITS1, B – ITS2).

Figure S4. The relative abundance (%) of OTUs of main fungal phyla in regions ITS1 (A) and ITS2 (B) in three tested biotopes.

Figure S5. The relative abundance (%) of OTUs of various fungal classes in regions ITS1 (A) and ITS2 (B) in three tested biotopes.

Figure S6: Maximum Likelihood phylogenetic tree that represents OTUs of *Ambispora* genus identified in ITS1 region,

Figure S7: ML phylogenetic tree that represents OTUs *Ambispora* genus identified in ITS2 region,

Figure S8: ML phylogenetic tree that represents OTUs of *Acaulospora* genus identified in ITS1 region,

Figure S9: ML phylogenetic tree that represents OTUs of *Acaulospora* genus identified in ITS2 region,

Figure S10: Dependencies between agrochemical parameters of rhizosphere soil and numbers of species and OTUs (for ITS1 region),

Figure S11: Dependencies between agrochemical parameters of rhizosphere soil and numbers of species and OTUs (for ITS2 region),

Table S4: Identification of arbuscular mycorrhizal fungi: species list according to OTUs founded with ITS1 analysis,

Table S5: Identification of arbuscular mycorrhizal fungi: genera list according to OTUs founded by ITS1 analysis,

Table S6: Identification of arbuscular mycorrhizal fungi: species list according to OTUs founded with ITS2 analysis,

Table S7: Identification of arbuscular mycorrhizal fungi: genera list according to OTUs founded by ITS2 analysis,

Table S8: List of common and endemicspecies (except VT) ranked in decreasing order of OTUs,

Table S9: Observed and extrapolated values of species richness, Shannon diversity, and Simpson diversity by OTUs,

Table S10. Advantages and disadvantages of molecular genetic methods using Illumina MiSeq to identify AMF species in comparison with morphological methods.

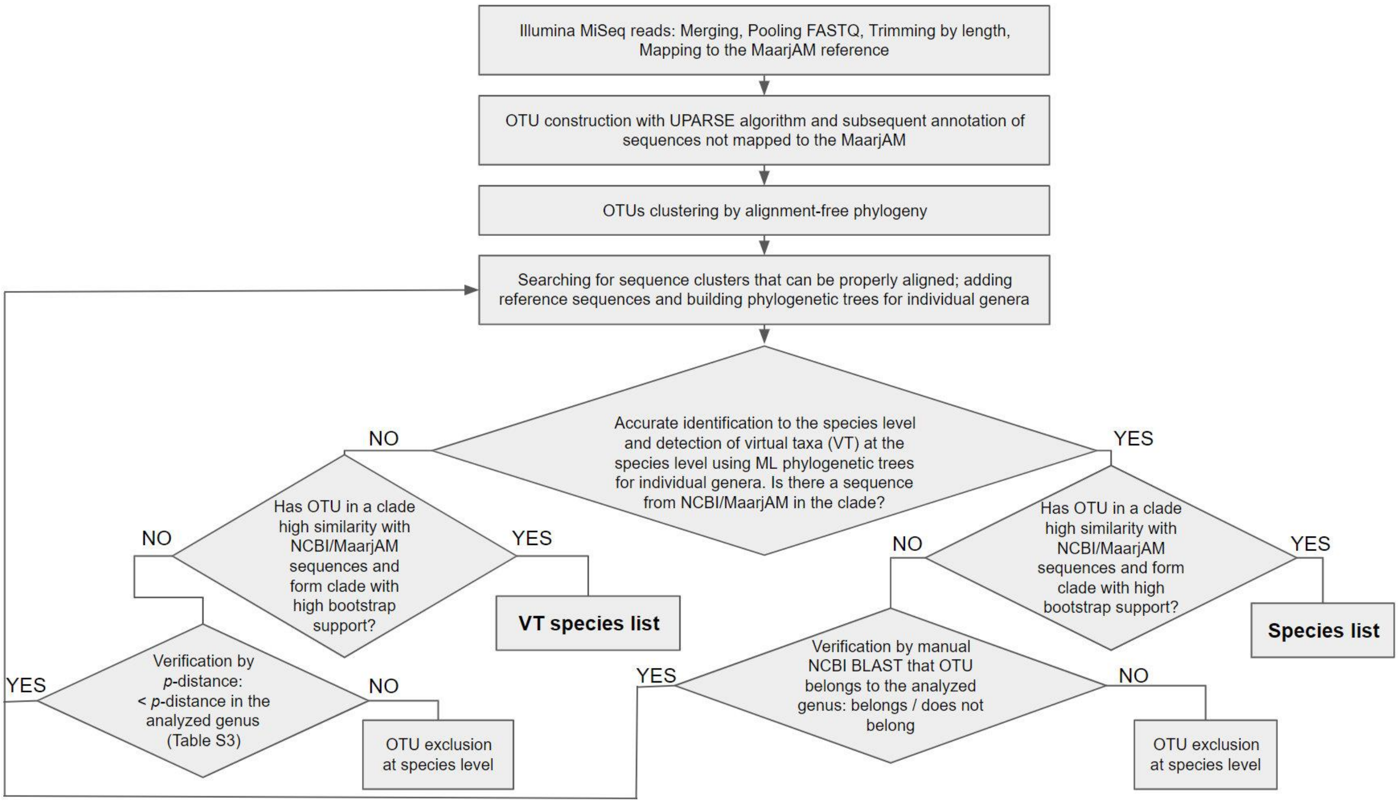

Figure S1. The algorithm for identification of AM fungi.

Table S1. Soil characteristics for analyzed stationary trial plots in the sampling horizon 0-10 cm.

Copyright: © 2023 by the authors. Licensee MDPI, Basel, Switzerland. This article is an open access article distributed under the terms and conditions of the Creative Commons Attribution (CC BY) license (https://creativecommons.org/licenses/by/4.0/).

STP Number	Stationary Trial Plot	pH_{KCl}	P_i, mg/kg	N_{total}, %	P_{total}, %	Sum of Fractions <0.01mm	Soil Type by Mechanical Composition
1	Subalpine Meadow-4, Malaya Hatipara ridge	5.87^{b}	9.7^{d}	0.64^{a}	0.08^{ab}	47.2^{a}	highly decomposed peat
3	Subalpine Meadow-3, Malaya Hatipara ridge	5.65^{b}	12.3^{d}	0.70^{a}	0.12^{a}	45.6^{a}	highly decomposed peat
4	Subalpine Meadow-2, Malaya Hatipara ridge	5.59^{b}	9.2^{d}	0.54^{ab}	0.08^{ab}	34.9^{b}	medium loam
7	Fir Forest-3, Malaya Hatipara mountain	3.82^{d}	7.2^{d}	0.30^{c}	0.04^{c}	22.2^{c}	light loam
8	Pine Forest-3, Malaya Hatipara mountain	3.92^{d}	17.7	0.45^{b}	0.06^{b}	40.6^{ab}	medium loam
9	Mixed forest near the Bolshaya Hatipara river, Bolshaya Hatipara mountain	4.45^{c}	54.5^{b}	0.67^{a}	0.11^{a}	17.2^{c}	heavy sandy loam
11	Grassland in the valley of the Teberda river, Teberda town	6.84^{a}	133.5^{a}	0.35^{c}	0.10^{a}	15.9^{c}	heavy sandy loam
12	Grassland in the valley of the Teberda river, the border of the New Teberda village	6.47^{a}	31.1^{c}	0.20^{d}	0.04^{c}	45.7^{a}	heavy loam
13	Grassland in the valley of the Kuban river, Ordzhonikidzevsky village	6.76^{a}	18.3^{cd}	0.18^{d}	0.07^{b}	18.1^{c}	heavy sandy loam

Note: P_i – inorganic phosphorus available for plant nutrition. Different letters (“a”, “b”, etc.) indicate significant differences within the same parameters (ANOVA and Tukey’s test; $P < 0.05$).

Table S2. Characteristics of stationary trial plots and plant communities.

STP Number	Stationary Trial (Sample) Plot	The Characteristics of Stationary Trial Plot	List of Plant Species	Number of Herbaceous Plant Species	Percent of Annual Plants
1	Subalpine Meadow-4, Malaya Hatipara ridge	Low-grass rocky subalpine meadow (the trial area – 10 m x 10 m) is located on the slope of a small mountain ridge, the high-altitude ecological profile in the Teberdinsky National Park. There are single Koch pine trees, rock outcrops occasionally. The surface of the soil is smooth, slightly sod-covered, there are many stones covered with lichens (from cobblestones to rubble). There are a lot of dry straw and dry fruits among the plants. The slope of the surface is 25° (very steep slope) with south-eastern exposure. Soil surface is from dry to moderately moist. Total projective coverage (TPC) is 80%.	Dominant species: cereals (*Agrostis capillaris, Bromopsis variegata, Elymus sp., Festuca rupicola*). Subdominant species: *Allium pseudostrictum*. Other species: *Achillea millefolium, Anthemis caucasica, Anthemis macroglossa, Campanula sp., Carduus adpressus, Chamaenerion angustifolium, Cotoneaster integerrimus, Dianthus ruprechtii, Gypsophila sp., Hieracium sp., Hypericum sp., Juniperus communis sbsp. hemisphaerica, Minuartia biebersteinii, Pimpinella saxifraga, Potentilla sp., Psephellus salvifolius, Pulsatilla sp., Rosa sp., Rumex sp., Scabiosa caucasica, Sedum spurium, Sempervivum caucasicum, Tragopogon reticulatus, Trifolium ambiguum, Trifolium sp.*	27	14%
3	Subalpine Meadow-3, Malaya Hatipara ridge	Subalpine Meadow-3, rocky high-grass cereal-mixed meadow (the trial area – 10 m x 10 m) is located on the slope of Malaya Hatipara ridge, the high-altitude ecological profile in the Teberdinsky National Park. There are cushions forming mosses and epilithic lichens on rocky ledges. The slope of the surface is 25-30° (very steep slope) with south-eastern exposure. Soil surface is moderately moist. TPC – 97%.	Dominant species: cereals (*Festuca sp., Agrostis planifolia*). Subdominant species: umbellifers (*Heracleum sp., Laserpitium stevenii, Bupleurum polyphyllum*). Other species: *Achillea millefolium, Achillea nobilis, Aconitum confertiflorum, Alchemilla minusculiflora, Anthemis caucasica, Anthemis macroglossa, Anthyllis vulneraria, Astrantia maxima, Briza elatior, Carduus adpressus, Cephalaria gigantea, Chamaenerion angustifolium, Dianthus ruprechtii, Erigeron venustus, Euphrasia hirtella, Fritillaria ophioglossifolia, Galium sp., Gentiana biebersteinii, Geranium ibericum, G. renardii, Gypsophila sp., Hedysarum caucasicum, Lapsana grandiflora, Lathyrus cyaneus, Linaria genistifolia, Linum hypericifolium, Lotus corniculatus, Myosotis alpestris, Pedicularis condensata, Phleum phleoides, Polygonum bistorta ssp. carneum, Polygonum panjutinii, Rhinanthus sp., Rhynchocorys orientalis, Rumex acetosa, Rumex alpinus, Scabiosa caucasica, Sedum spurium,*	50	10%

			Sempervivum caucasicum, Solidago virgaurea, Stachys macrantha, Taraxacum sp., Trifolium ambiguum, Trifolium polyphyllum, Vicia tenuifolia ssp. subalpinus. There are rare low trees and shrubs around: *Pinus sylvestris ssp. hamata, Betula pendula, Salix sp., Juniperus communis ssp. hemisphaerica, Rosa sp.*		
4	Subalpine Meadow-2, Malaya Hatipara ridge	Subalpine Meadow-2, high-grass mixed-grass meadow (the trial area – 10 m x 10 m) is located on the slope of Malaya Hatipara ridge, the high-altitude ecological profile in the Teberdinsky National Park. Sparse trees areas with a predominance of pine are above and below the slope. Soil surface is sod-covered, hummocky. The slope of the surface is 20° (very steep slope) with south-eastern exposure. Soil surface is from dry to moderately moist. TPC – 95%.	Dominant species: umbellifers (*Agasyllis latifolia, Angelica tatianae, Anthriscus sylvestris, Bupleurum polyphyllum, Heracleum sp., Pimpinella rhodantha, P. saxifraga*). Subdominant species: *Chamaenerion angustifolium*, *Trisetum flavescens*. Other species: *Achillea millefolium, Aconitum confertiflorum, A. cymbulatum, A. orientale, Agrostis capillaris, Alchemilla minusculiflora, Astragalus sp., Campanula latifolia, Carduus adpressus, Centaurea abbreviata, Cephalaria gigantea, Cirsium obvallatum, Dactylus glomerata, Digitalis ciliata, Festuca sp., Galium valantioides, Gentiana septemfida, Geranium sp., Hieracium sp., Lapsana grandiflora, Latyrus pratensis, Leonurus quinquelobatus, Milium effusum, Oberna behen, Phleum sp., Polygonum bistorta ssp. carneum, Ranunculus caucasicus ssp. subleiocarpus, Rubus idaeus, Rumex sp., Scabiosa caucasica, Sedum spurium, Senecio jacquinianus, Stachys macrantha, Trifolium ambiguum, Urtica dioica, Veratrum album, Vicia sepium, V. tenuifolia ssp. subalpinus*.	46	4%
7	Fir Forest-3, Malaya Hatipara mountain	Fir Forest-3, dead-cover moist green-moss fir forest (*Abies nordmanniana*; the trial area – 10 m x 10 m) is located on the slope of Malaya Hatipara ridge, the high-altitude ecological profile in the Teberdinsky National Park. The slope of the surface is 44° (very steep slope) with northern exposure. Projective coverage (PC) of the lower storey is 20%.	Herbaceous plant species: *Festuca altissima, Gymnocarpium dryopteris, Oxalis acetosella, Polygonatum orientale, Polypodium vulgare, Senecio renifolius, Solidago virgaurea, Trifolium sp.,Valeriana alliariifolia.*Mosses species were not determined.	9	10%
8	Pine Forest-3, Malaya Hatipara mountain	Pine Forest-3, pine (*Pinus sylvestris*) with reed grass (the trial area – 10 m x 10 m) is located on the slope of Malaya Hatipara ridge, the high-altitude ecological profile in the Teberdinsky	Dominant species: *Calamagrostis arundinacea, Digitalis ciliata*. Other species:*Asyneuma campanuloides, Campanula rapunculoides, Festuca altissima, Trifolium sp.*	6	14%

		National Park. The slope of the surface is 43° (very steep slope) with southern exposure. Soil surface is dry. PC of the lower storey is 15%.			
9	Mixed forest near the Bolshaya Hatipara river, Bolshaya Hatipara mountain	The main tree storey of mixed forest is *Betula pendula*, *Fagus orientalis*, *Picea orientalis*, the underwood – *Acer sp.*, *Sorbus aucuparia*, *Corylus avellana*, the young growth – *Abies nordmanniana*. The trial area (10 m x 10 m) is a glade in the forest, the high-altitude ecological profile in the Teberdinsky National Park. In the lower storey there are mosses in places. Forest litter is mainly represented by leaf litter. The slope of the surface is 10° (steep slope) towards the river. Soil surface is moderately moist. PC of the lower storey is 75%.	Dominant species: *Petasites albus*. Subdominant species: *Brachypodium pinnatum*, *Aconitum nasutum*. Other species: *Anthriscus sylvestris, Calamagrostis arundinacea, Calamintha grandiflora, Fragaria vesca, Galium odoratum, Geranium robertianum, Melica nutans, Mycelis muralis, Oxalis acetosella, Polygonatum verticulatum, Salvia glutinosa, Telekia speciosa, Thalictrum sp., Trifolium sp., Vicia sepium.*	18	10%
11	Grassland in the valley of the Teberda river, Teberda town	Disturbed grassland (the trial area – 10 m x 10 m) is located in the river valley (second terrace), the Teberdinsky National Park. The soil surface is smooth with almost no slope (the surface is 2-3°, flat slope) towards the river., with stones and boulders covered with lichens. Soil surface is moderately moist. TPC – 80%.	Dominant species: *Trifolium repens.* Subdominant species: *Setaria pumila.* Other species:*Achillea millefolium,Alchemilla tytthantha, Lolium perenne, Medicago falcata, M. lupulina, Plantago major, Polygonum neglectum, Rumex acetosella, Sedum pallidum, Taraxacum officinale, Trifolium campestre, Vicia sepium*.	14	31%
12	Grassland in the valley of the Teberda river, the border of the New Teberda village	Disturbed grassland (the trial area – 10 m x 10 m) is located in the river valley (second terrace) surrounded by deciduous trees and shrubs (*Corylus avellana, Crataegus monogyna, Prunus sp., Ulmus minor*). The soil surface is smooth with almost no slope (the surface is 1-3°, flat slope). Soil surface is from dry to moderately moist. TPC – 80%.	Dominant species: *Urtica dioica, Fragaria vesca*. Subdominant species: cereals (*Holcus lanatus, Bromus arvensis, Echinochloa crus-galli*). Other species:*Achillea millefolium, Agrimonia pilosa, Alchemilla caucasica, Arctium lappula, Carduus nutans, Cichorium intybus, Cirsium arvense, Convolvulus arvensis, Conyza canadensis, Dactylus glomerata, Daucus carota, Echium vulgare, Erigeron acris, Fragaria vesca, Holcus lanatus, Galega orientalis, Galeopsis tetrahit, Galinsoga parviflora, Glehoma hederacea, Leontodon hispidus, Lotus corniculatus, Medicago lupulina, Origanum vulgare, Phalacroloma annuum, Plantago lanceolata, P. major, Polygonum lapathifolium, Prunella vulgaris, Raphanus raphanistrum, Rosa canina, Salvia*	44	24%

			verticillata, Sedum spurium, Setaria pumila, Sonchus arvensis, Taraxacum officinale, Trifolium pratense, T. repens, Triticum aestivum, Vicia tenuifolia, Vicia sp.		
13	Grassland in the valley of the Kuban river, Ordzhonikidzevsky village	Disturbed floodplain grassland (the trial area – 10 m x 10 m) is located in the river valley (second terrace), fresh ruderal meadow. Small bushes of *Alnus glutinosa*. The soil surface is sod-formed, bumpy without slope (the surface is 1-2°, flat slope). Soil surface is from dry to moderately moist. TPC – 100%.	Dominant species: *Ambrosia artemisiifolia*. Subdominant species: *Setaria pumila*. Other species: *Achillea millefolium, Artemisia absintium, Astragalus sp., Berteroa incana, Centaurea biebersteinii, Cichorium intybus, Cirsium sp., Convolvulus arvensis, Elytrigia repens, Eurodium cicutarium, Galium humifusum, Lotus corniculatus, Medicago falcata, M. lupulina, Plantago lanceolata, Potentilla adscharica, Reseda lutea, Taraxacum officinale, Trifolium arvense, T. campestre, T. pratense, T. repens, Vicia cracca, Xanthium albinum.*	26	28%

Table S3. Average and largest p-distances in ITS1 and ITS2 regions for various AMF genera with more than one species.

AMF genus	Average *p*-distance value		Maximum *p*-distance value	
	ITS1	ITS2	ITS1	ITS2
Acaulospora	0.39	0.24	0.60	0.44
Ambispora	0.20	0.15	0.42	0.32
Archaeospora	0.27	0.24	0.41	0.36
Cetratospora	0.17	0.15	0.26	0.28
Claroideoglomus	0.19	0.24	0.35	0.40
Corymbiglomus	0.27	0.21	0.30	0.25
Dentiscutata	0.09	0.11	0.21	0.16
Diversispora	0.14	0.12	0.26	0.23
Dominikia	0.27	0.18	0.46	0.29
*Entrophospora**	0.10	0.12	0.14	0.17
Funneliformis	0.17	0.12	0.42	0.23
Gigaspora	0.11	0.10	0.27	0.18
Glomus	0.37	0.37	0.50	0.61
*Innospora**	0.01	0.01	0.01	0.01
Kamienskia	0.32	0.30	0.37	0.39
*Oehlia**	0.26	0.23	0.38	0.31
Paraglomus	0.30	0.24	0.36	0.30
*Pervetustus**	0.03	0.05	0.03	0.05
Racocetra	0.14	0.11	0.28	0.18
Redeckera	0.31	0.13	0.39	0.19
Rhizoglomus	0.15	0.19	0.18	0.25
Rhizophagus	0.20	0.19	0.47	0.40
Sacculospora	0.27	0.14	0.41	0.21
*Sclerocarpum**	0.11	0.07	0.13	0.11
Sculellospora	0.21	0.18	0.27	0.24
Septoglomus	0.16	0.20	0.33	0.36

Note: *genus with one species (monotypic genus).

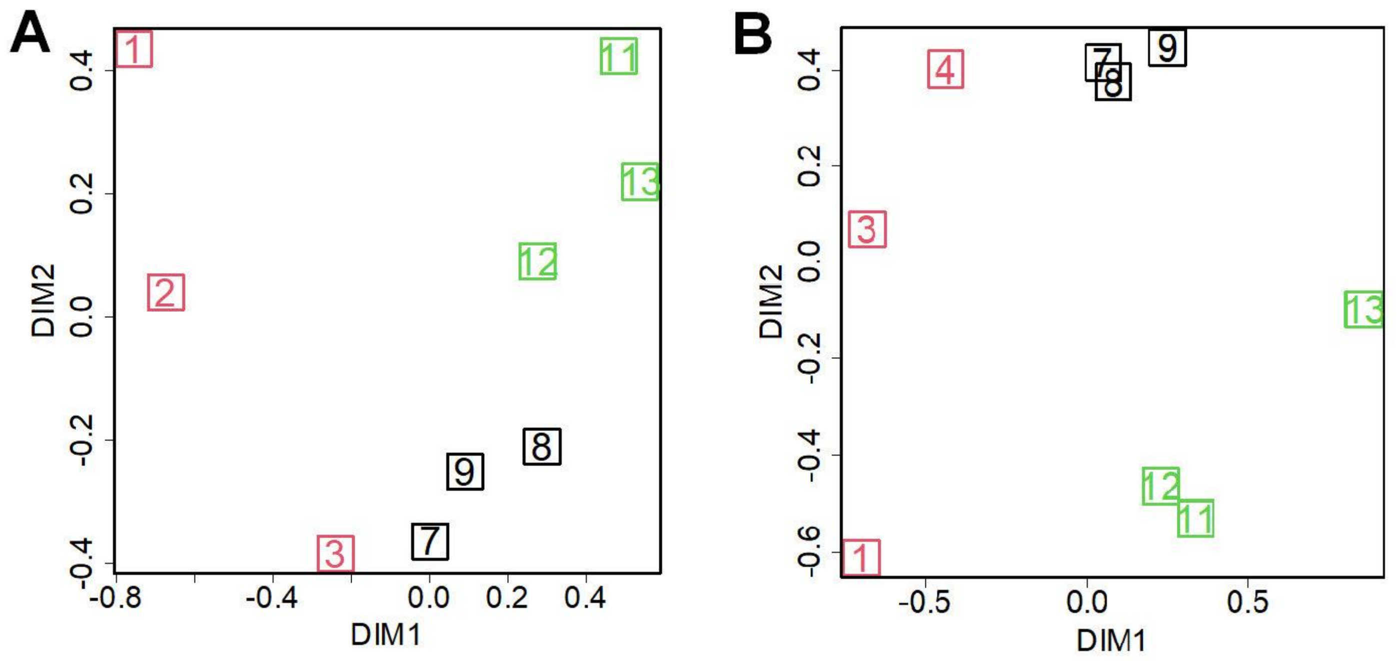

Figure S2. Samples representation in low dimensional space revealed from MDS (Multidimesional scaling). Spearman's distance (1-rho) was used as metric of dissimilarity between OTUs profiles (A – ITS1, B - ITS2). Numbers in squares are STPs where soil samples were taken, colors red – subalpine meadow, black – forest, green – river valley.

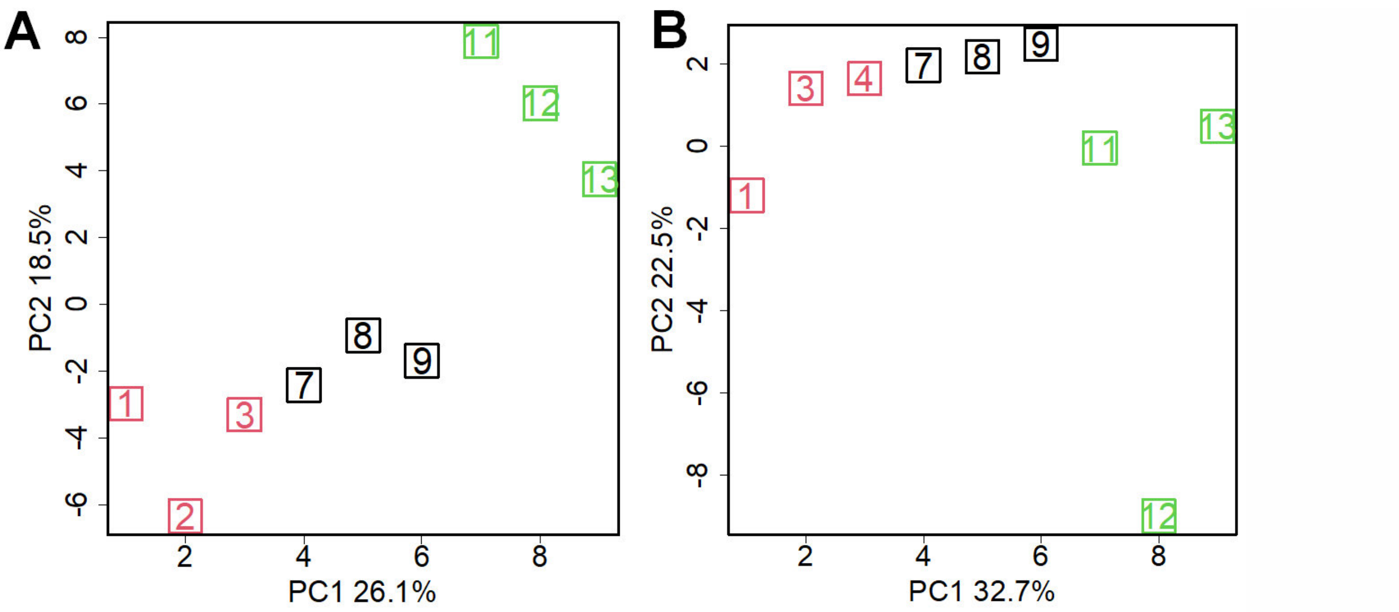

Figure S3. Score plots from PCA of OTUs profiles (A – ITS1, B – ITS2). Data were normalized per sample sum and autoscaled. Numbers in squares are STPs where soil samples were taken, colors red – subalpine meadow, black – forest, green – river valley.

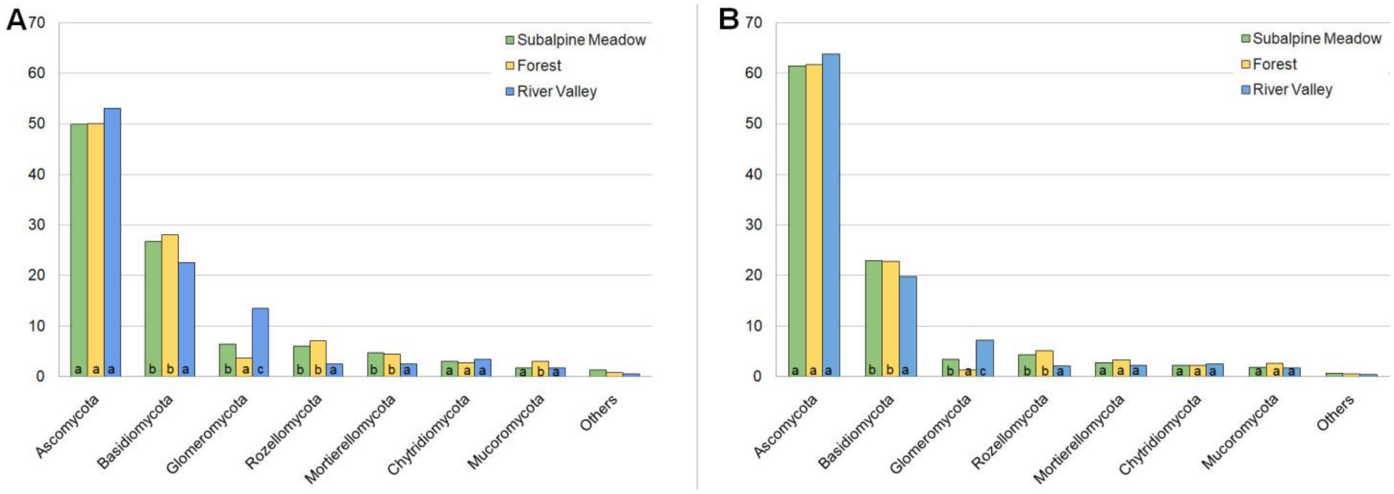

Figure S4. The relative abundance (%) of OTUs of main fungal phyla in regions ITS1 (A) and ITS2 (B) in three tested biotopes. Different letters (a, b, c) indicate significant differences in the relative abundance of OTUs within the same fungal phylum (ANOVA; $p < 0.05$)

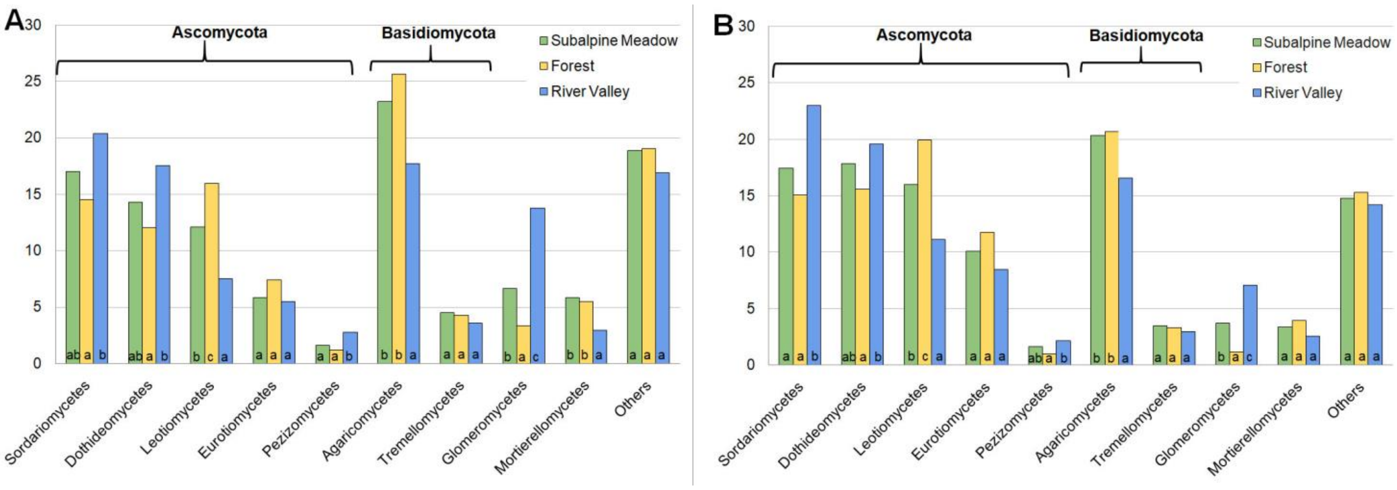

Figure S5. The relative abundance (%) of OTUs of various fungal classes in regions ITS1 (A) and ITS2 (B) in three tested biotopes. Different letters (a, b, c) indicate significant differences in the relative abundance of OTUs within the same fungal class (ANOVA; $p < 0.05$)

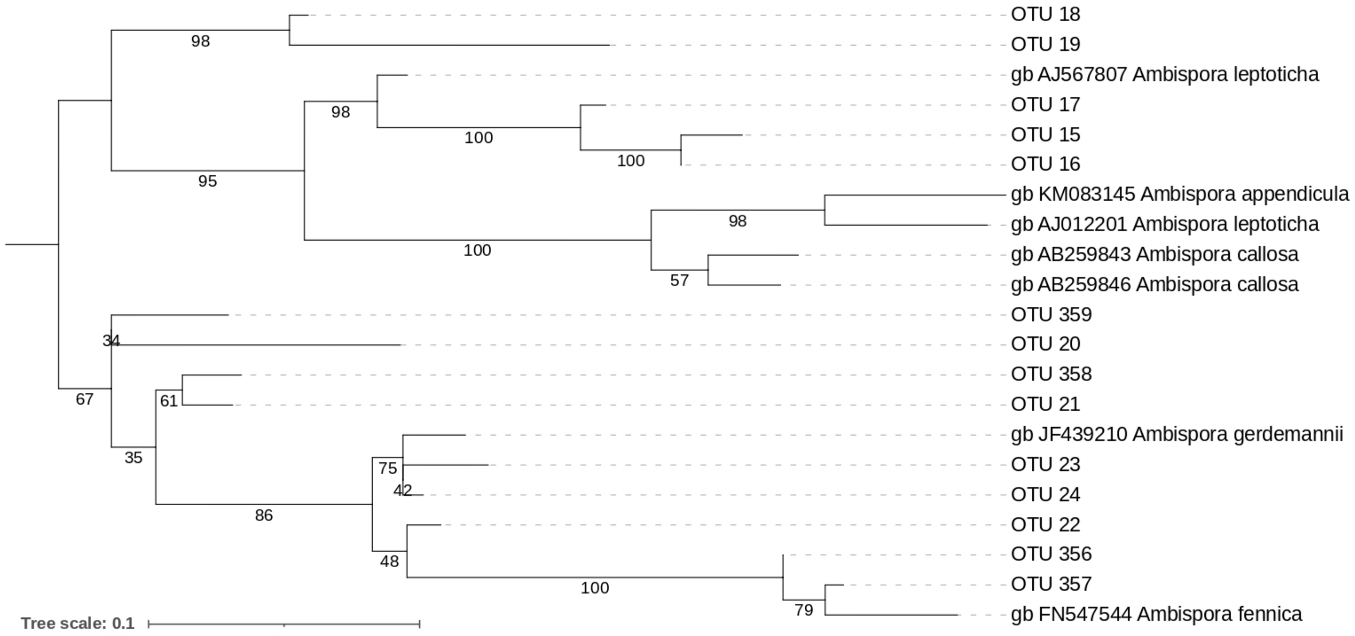

Figure S6. Maximum Likelihood phylogenetic tree built that represent OTUs of *Ambispora* genus identified for ITS1 region. "OTU" – operational taxonomic units obtained in this study; "gb" – sequences from NCBI GenBank.

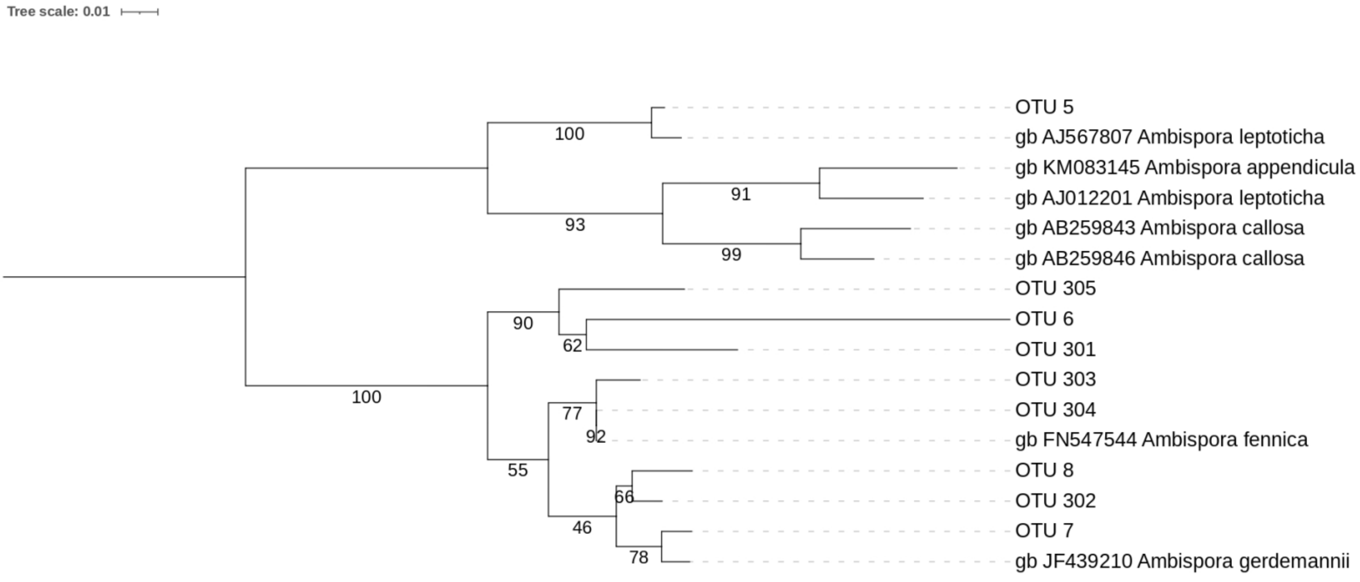

Figure S7. ML phylogenetic tree built that represent OTUs of *Ambispora* genus identified for ITS2 region.

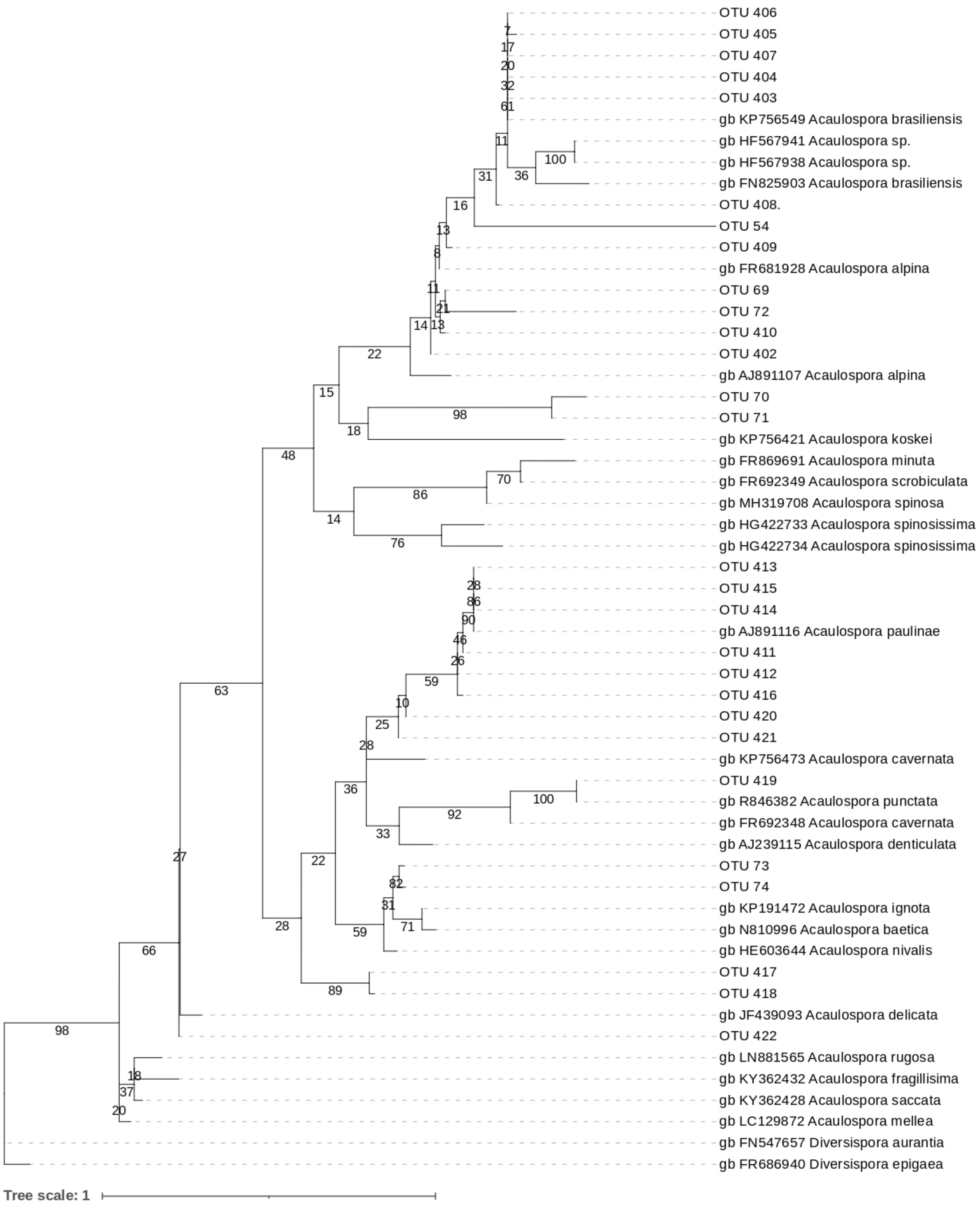

Figure S8. ML phylogenetic tree built that represent OTUs of *Acaulospora* genus identified for ITS1 region.

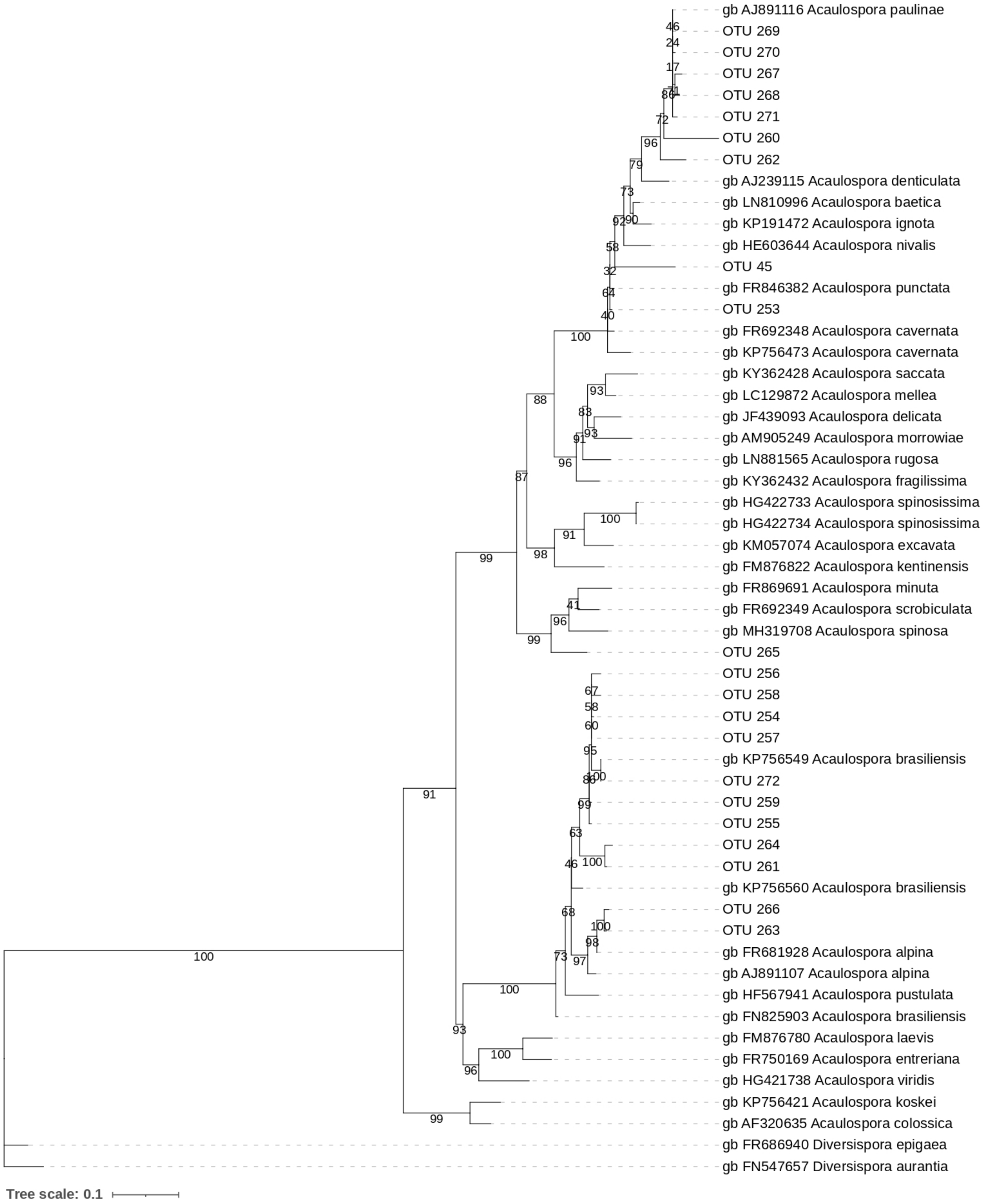

Figure S9. ML phylogenetic tree built that represent OTUs of *Acaulospora* genus identified for ITS2 region.

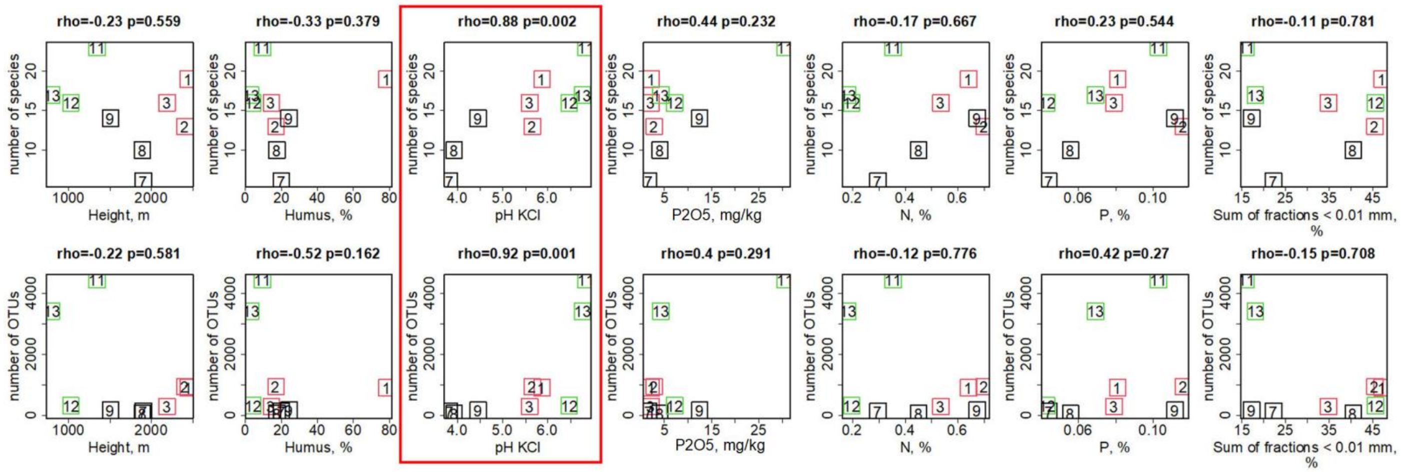

Figure S10. Dependencies between agrochemical parameters of rhizosphere soil and numbers of species and OTUs (for ITS1 region). Hight is altitude above sea level, rho – Spearman correlation, N – number of OTUs, the numbers in the squares are the numbers of the spots where the samples were taken, colors: red – meadow, black – forest, green – valley, "P2O5" – P_2O_5 (mg/kg; mobile form of inorganic phosphorus available for plant nutrition). Spearman correlation between the number of OTUs for ITS1 with height (altitude) and agrochemical parameters of rhizospheric soil from which the samples were taken.

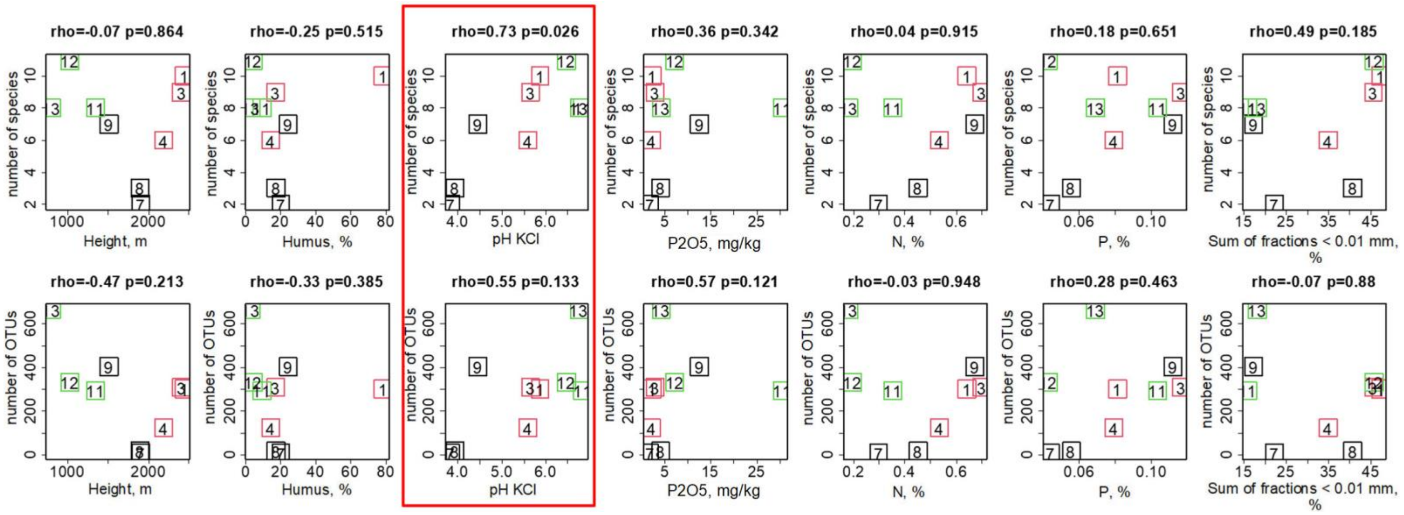

Figure S11. Dependencies between agrochemical parameters of rhizosphere soil and numbers of species and OTUs (for ITS2 region). Hight is altitude above sea level, rho – Spearman correlation, N – number of OTUs, the numbers in the squares are the numbers of the spots where the samples were taken, colors: red – meadow, black – forest, green – valley, "P2O5" – P_2O_5 (mg/kg; mobile form of inorganic phosphorus available for plant nutrition). Spearman correlation between the number of OTUs for ITS2 with height (altitude) and agrochemical parameters of rhizospheric soil from which the samples were taken.

Table S4. Identification of arbuscular mycorrhizal fungi: species list according to OTUs founded by ITS1 analysis.

Number of OTUs for ITS1 / Species list for ITS1	OTUs for meadow biotopes			OTUs for forest biotopes			OTUs for river valley (grassland, disturbed area) biotopes			Total	Total OTUs for			Total % of OTUs for			Total %
	STP #1	STP #3	STP #4	STP #7	STP #8	STP #9	STP #11	STP #12	STP #13	OTUs	Meadow	Forest	Valley	Meadow	Forest	Valley	of OTUs
Acaulospora delicata	0	0	0	0	0	0	1	0	0	1	0	0	1	0.0	0.0	0.5	0.3
Diversispora slowinskiensis	0	0	0	0	0	1	0	0	0	1	0	1	0	0.0	1.2	0.0	0.3
Diversispora spurca	0	0	0	0	0	1	0	0	0	1	0	1	0	0.0	1.2	0.0	0.3
Diversispora varaderana	0	0	0	0	0	0	0	0	1	1	0	0	1	0.0	0.0	0.5	0.3
Dominikia achra	0	0	0	0	0	0	1	0	0	1	0	0	1	0.0	0.0	0.5	0.3
Halonatospora pansihalos	0	0	0	0	0	0	0	0	1	1	0	0	1	0.0	0.0	0.5	0.3
Acaulospora punctata	0	0	1	0	0	1	0	0	0	1	1	1	0	0.9	1.2	0.0	0.3
Acaulospora viridis	0	0	1	0	0	0	0	0	0	1	1	0	0	0.9	0.0	0.0	0.3
Archaeospora europaea	0	1	0	0	0	0	1	0	0	1	1	0	1	0.9	0.0	0.5	0.3
Archaeospora trappei	1	1	0	0	0	0	1	0	0	1	1	0	1	0.9	0.0	0.5	0.3
Paraglomus brasilianum	0	0	1	0	0	0	0	0	0	1	1	0	0	0.9	0.0	0.0	0.3
Ambispora fennica	0	0	0	0	1	0	2	0	0	2	0	1	2	0.0	1.2	0.9	0.7
Claroideoglomus lamellosum	0	0	0	0	0	0	2	0	0	2	0	0	2	0.0	0.0	0.9	0.7
Diversispora celata	0	0	0	0	0	0	0	1	1	2	0	0	1	0.0	0.0	0.5	0.7
Diversispora sporocarpia	0	0	0	0	0	2	0	0	0	2	0	2	0	0.0	2.4	0.0	0.7
Palaeospora spainii	0	0	0	0	0	0	2	0	0	2	0	0	2	0.0	0.0	0.9	0.7
Ambispora gerdemannii	1	0	0	0	1	0	0	0	2	2	1	2	2	0.9	2.4	0.9	0.7
Diversispora insculpta	1	1	0	1	0	0	0	0	0	2	1	1	0	0.9	1.2	0.0	0.7
Acaulospora nivalis	0	0	2	0	1	0	0	0	0	2	2	1	0	1.8	1.2	0.0	0.7
Septoglomus viscosum	0	0	0	0	0	0	0	0	3	3	0	0	3	0.0	0.0	1.4	**1.0**
Ambispora leptoticha	0	0	1	0	0	3	0	0	0	3	1	3	0	0.9	3.5	0.0	**1.0**
Glomus macrocarpum	1	1	1	0	0	1	2	0	0	3	1	1	2	0.9	1.2	0.9	**1.0**
Septoglomus nigrum	1	0	1	0	0	3	2	2	0	3	1	3	3	0.9	3.5	1.4	**1.0**
Otospora bareae	1	1	0	0	0	1	1	0	2	3	2	1	3	1.8	1.2	1.4	**1.0**
Acaulospora alpina	0	0	3	0	0	0	0	0	0	3	3	0	0	2.7	0.0	0.0	**1.0**
Claroideoglomus walkeri	1	0	0	0	0	0	5	0	2	6	1	0	6	0.9	0.0	2.8	**2.1**
Dominikia disticha	1	0	0	0	0	0	6	0	0	6	1	0	6	0.9	0.0	2.8	**2.1**
Rhizoglomus invermaium	0	0	0	0	1	0	4	0	4	7	0	1	6	0.0	1.2	2.8	**2.4**
Funneliformis mosseae	0	0	0	0	0	0	8	0	0	8	0	0	8	0.0	0.0	3.7	**2.8**
Septoglomus constrictum	2	0	1	0	4	2	3	3	4	8	2	5	7	1.8	5.9	3.3	**2.8**
Acaulospora paulinae	1	0	0	0	1	6	7	0	0	9	1	6	7	0.9	7.1	3.3	**3.1**
Glomus indicum	6	0	1	0	0	0	7	5	7	12	6	1	10	5.4	1.2	4.7	**4.2**
Glomus tetrastratosum	5	2	3	2	0	0	3	0	0	12	8	2	3	7.2	2.4	1.4	**4.2**
Claroideoglomus claroideum	3	8	1	0	0	1	7	1	2	12	9	1	8	8.1	1.2	3.7	**4.2**
Rhizophagus irregularis	3	3	4	1	1	9	8	4	10	17	6	9	15	5.4	10.6	7.0	**5.9**
Paraglomus laccatum	2	0	3	2	0	6	19	3	6	22	4	8	19	3.6	9.4	8.8	**7.7**
Rhizophagus intraradices	10	7	5	3	3	9	15	3	8	24	10	13	21	9.0	15.3	9.8	**8.4**
Dominikia bernensis	8	1	3	1	1	1	13	4	5	26	11	3	19	9.9	3.5	8.8	**9.1**
VT03 *Archaeospora sp.*	1	0	0	0	0	0	0	0	0	1	1	0	0	0.9	0.0	0.0	0.3
VT01 *Acaulospora sp.*	1	0	2	0	0	0	0	0	0	2	2	0	0	1.8	0.0	0.0	0.7
VT02 *Acaulospora sp.*	0	0	2	0	0	0	2	0	0	2	2	1	2	1.8	1.2	0.9	0.7
VT04 *Archaeospora sp.*	0	1	0	0	0	0	1	0	0	2	1	0	1	0.9	0.0	0.5	0.7
VT06 *Archaeospora sp.*	0	0	0	0	0	0	2	0	0	2	0	0	2	0.0	0.0	0.9	0.7
VT07 *Diversispora sp.*	0	0	0	0	0	0	2	0	0	2	0	0	2	0.0	0.0	0.9	0.7
VT09 *Diversispora sp.*	0	0	0	0	0	0	2	0	0	2	0	0	2	0.0	0.0	0.9	0.7
VT10 *Scutellospora sp.*	0	0	1	0	0	1	1	1	0	2	1	1	1	0.9	1.2	0.5	0.7
VT12 *Rhizophagus sp.*	0	0	0	0	1	0	0	1	2	2	0	1	2	0.0	1.2	0.9	0.7
VT14 *Rhizophagus sp.*	2	0	0	0	0	0	0	0	0	2	2	0	0	1.8	0.0	0.0	0.7
VT17 *Rhizophagus sp.*	1	0	1	0	0	0	0	0	1	2	2	0	1	1.8	0.0	0.5	0.7
VT18 *Dominikia sp.*	0	0	0	0	0	0	0	2	0	2	0	0	2	0.0	0.0	0.9	0.7
VT19 *Claroideoglomus sp.*	2	2	0	0	0	0	1	0	0	2	2	0	1	1.8	0.0	0.5	0.7
VT23 *Rhizophagus sp.*	1	0	1	0	1	2	2	1	1	2	1	2	2	0.9	2.4	0.9	0.7
VT24 *Rhizophagus sp.*	1	0	1	0	0	1	1	1	0	2	1	1	2	0.9	1.2	0.9	0.7
VT25 *Rhizophagus sp.*	1	1	1	1	1	2	2	0	0	2	1	2	2	0.9	2.4	0.9	0.7
**VT05 *Archaeospora sp.***	0	0	1	0	0	1	2	0	0	3	1	1	2	0.9	1.2	0.9	**1.0**
**VT08 *Diversispora sp.***	0	0	0	0	1	0	1	0	3	3	0	1	3	0.0	1.2	1.4	**1.0**
**VT11 *Rhizophagus sp.***	0	0	0	0	0	0	1	1	3	3	0	0	3	0.0	0.0	1.4	**1.0**
**VT13 *Rhizophagus sp.***	2	1	2	1	0	0	1	0	1	3	2	1	2	1.8	1.2	0.9	**1.0**
**VT20 *Dominikia sp.***	0	0	2	0	0	0	2	0	0	3	2	0	2	1.8	0.0	0.9	**1.0**
**VT21 *Septoglomus sp.***	0	0	0	0	0	0	0	0	3	3	0	0	3	0.0	0.0	1.4	**1.0**
**VT26 *Glomus sp.***	2	1	3	0	0	0	2	0	0	3	3	0	2	2.7	0.0	0.9	**1.0**
**VT15 *Rhizophagus sp.***	3	1	4	3	0	0	1	0	0	4	4	3	1	3.6	3.5	0.5	**1.4**
**VT16 *Rhizophagus sp.***	4	3	1	0	1	1	0	0	1	4	4	3	1	3.6	3.5	0.5	**1.4**
**VT22 *Rhizophagus sp.***	1	0	0	0	0	0	4	1	0	5	2	0	5	1.8	0.0	2.3	**1.7**
**VT27 *Dominikia sp.***	0	0	1	0	1	0	0	4	8	8	1	1	8	0.9	1.2	3.7	**2.8**
Total OTUs:	70	36	55	15	20	55	150	38	81	287	111	85	215				

Note: the species with total rate of OTUs >1% are highlighted in bold.

Table S5. Identification of arbuscular mycorrhizal fungi: genera list according to OTUs founded by ITS1 analysis.

Number of OTUs for ITS1	OTUs for meadow biotopes			OTUs for forest biotopes			OTUs for river valley (grassland, disturbed area) biotopes			Total	Total OTUs for			Total % of OTUs for			Total %
Species list for ITS1	STP #1	STP #3	STP #4	STP #7	STP #8	STP #9	STP #11	STP #12	STP #13	OTUs	Meadow	Forest	Valley	Meadow	Forest	Valley	of OTUs
Rhizophagus	29	16	20	9	9	24	39	12	31	79	35	36	63	31.5	42.4	29.3	27.5
Dominikia	9	1	6	1	2	1	22	10	13	46	15	4	38	13.5	4.7	17.7	16.0
Glomus	14	4	8	2	0	1	14	5	7	30	18	4	17	16.2	4.7	7.9	10.5
Paraglomus	2	0	4	2	0	6	19	3	6	23	5	8	19	4.5	9.4	8.8	8.0
Claroideoglomus	6	10	1	0	0	1	15	1	4	22	12	1	17	10.8	1.2	7.9	7.7
Acaulospora	2	0	11	0	2	7	10	0	0	21	12	9	10	10.8	10.6	4.7	7.3
Septoglomus	3	0	2	0	4	5	5	5	10	17	3	8	16	2.7	9.4	7.4	5.9
Diversispora	1	1	0	1	1	4	5	1	5	16	1	6	9	0.9	7.1	4.2	5.6
Archaeospora	2	3	1	0	0	1	7	0	0	10	5	1	7	4.5	1.2	3.3	3.5
Funneliformis	0	0	0	0	0	0	8	0	0	8	0	0	8	0.0	0.0	3.7	2.8
Ambispora	1	0	1	0	2	3	2	0	2	7	2	6	4	1.8	7.1	1.9	2.4
Otospora	1	1	0	0	0	1	1	0	2	3	2	1	3	1.8	1.2	1.4	1.0
Scutellospora	0	0	1	0	0	1	1	1	0	2	1	1	1	0.9	1.2	0.5	0.7
Palaeospora	0	0	0	0	0	0	2	0	0	2	0	0	2	0.0	0.0	0.9	0.7
Halonatospora	0	0	0	0	0	0	0	0	1	1	0	0	1	0.0	0.0	0.5	0.3
Total OTUs:	70	36	55	15	20	55	150	38	81	287	111	85	215				

Table S6. Identification of arbuscular mycorrhizal fungi: species list according to OTUs founded by ITS2 analysis.

Number of OTUs for ITS2	OTUs for meadow biotopes			OTUs for forest biotopes			OTUs for river valley (grassland, disturbed area) biotopes			Total	Total OTUs for			Total % of OTUs for			Total %
Species list for ITS2	STP #1	STP #3	STP #4	STP #7	STP #8	STP #9	STP #11	STP #12	STP #13	OTUs	Meadow	Forest	Valley	Meadow	Forest	Valley	of OTUs
Acaulospora punctata	0	0	0	0	0	0	1	0	0	1	0	0	1	0.0	0.0	0.5	0.4
Ambispora leptoticha	0	0	1	0	0	1	0	0	0	1	1	1	0	0.8	1.8	0.0	0.4
Cetraspora gilmorei	0	0	0	0	0	0	0	0	1	1	0	0	1	0.0	0.0	0.5	0.4
Claroideoglomus hanlinii	0	0	0	0	0	0	0	0	1	1	0	0	1	0.0	0.0	0.5	0.4
Diversispora slowinskensis	0	0	0	0	0	1	0	0	0	1	0	1	0	0.0	1.8	0.0	0.4
Diversispora varaderana	1	0	0	0	0	0	0	0	0	1	1	0	0	0.8	0.0	0.0	0.4
Dominikia difficilevidera	0	0	0	0	0	0	0	1	0	1	0	0	1	0.0	0.0	0.5	0.4
Glomus bareae	1	0	0	0	0	0	0	0	0	1	1	0	0	0.8	0.0	0.0	0.4
Glomus hoi	0	0	0	0	0	1	1	1	1	1	0	1	1	0.0	1.8	0.5	0.4
Halonatospora pansihalos	0	0	0	0	0	0	0	0	6	1	0	0	1	0.0	0.0	0.5	0.4
Scutellospora alterata	0	0	0	0	0	6	0	0	0	1	0	1	0	0.0	1.8	0.0	0.4
Scutellospora pellucida	0	0	0	0	0	0	0	1	0	1	0	0	1	0.0	0.0	0.5	0.4
Acaulospora alpina	0	0	2	0	0	0	0	0	0	2	2	0	0	1.7	0.0	0.0	0.7
Ambispora fennica	0	0	0	0	0	0	2	0	0	2	0	0	2	0.0	0.0	1.0	0.7
Ambispora gerdemannii	1	0	0	0	0	1	0	1	1	2	1	1	1	0.8	1.8	0.5	0.7
Archaeospora spainiae	0	0	0	0	0	0	2	0	0	2	0	0	2	0.0	0.0	1.0	0.7
Glomus indicum	2	1	0	0	0	1	1	1	0	2	2	1	1	1.7	1.8	0.5	0.7
Glomus macrocarpum	0	1	1	0	0	0	0	0	1	2	2	0	1	1.7	0.0	0.5	0.7
Rhizophagus aggregatus	0	0	0	0	0	0	0	1	1	2	0	0	2	0.0	0.0	1.0	0.7
Rhizoglomus melanus	3	1	1	0	0	0	0	0	0	3	3	0	0	2.5	0.0	0.0	1.1
Archaeospora trappei	0	3	0	0	0	0	6	0	1	6	3	0	6	2.5	0.0	3.0	**2.2**
Dominikia bernensis	3	0	0	0	0	0	5	1	4	6	3	0	6	2.5	0.0	3.0	**2.2**
Rhizophagus invermaius	8	0	0	0	0	0	11	0	71	6	1	0	5	0.8	0.0	2.5	**2.2**
Acaulospora paulinae	2	1	0	0	0	4	5	0	0	7	2	4	5	1.7	7.1	2.5	**2.6**
Claroideoglomus lamellosum	8	1	0	0	0	1	29	11	13	7	2	1	7	1.7	1.8	3.6	**2.6**
Entrophospora infrequens	3	4	3	0	0	2	2	5	4	7	4	2	5	3.4	3.6	2.5	**2.6**
Acaulospora brasiliensis	0	0	9	0	0	0	0	0	0	9	9	0	0	7.6	0.0	0.0	**3.3**
Claroideoglomus claroideum	4	7	8	0	0	2	4	6	2	9	8	3	6	6.7	5.4	3.0	**3.3**
Funneliformis mosseae	0	0	0	0	0	0	7	2	0	9	0	0	9	0.0	0.0	4.6	**3.3**
Paraglomus laccatum	1	1	2	0	1	2	14	4	2	15	3	2	15	2.5	3.6	7.6	**5.5**
Rhizophagus irregularis	2	4	4	0	2	9	9	6	7	16	7	9	13	5.9	16.1	6.6	**5.9**
Rhizophagus intraradices	7	8	6	1	3	13	12	5	13	27	14	13	20	11.8	23.2	10.2	**9.9**
VT28 *Archaeospora sp.*	0	0	0	0	0	0	1	0	0	1	0	0	1	0.0	0.0	0.5	0.4
VT29 *Claroideoglomus sp.*	0	0	0	0	0	0	1	1	0	1	0	0	1	0.0	0.0	0.5	0.4
VT31 *Glomus sp.*	0	0	1	0	0	0	0	0	0	1	1	0	0	0.8	0.0	0.0	0.4
VT33 *Glomus sp.*	1	1	1	1	0	0	0	0	0	2	2	1	0	1.7	1.8	0.0	0.7
VT41 *Glomus sp.*	1	1	2	0	0	0	1	1	0	2	2	0	1	1.7	0.0	0.5	0.7
VT43 *Archaeospora sp.*	0	0	0	0	0	0	2	0	0	2	0	0	2	0.0	0.0	1.0	0.7
VT49 *Scutellospora sp.*	0	0	2	0	0	1	0	1	0	2	1	1	1	0.8	1.8	0.5	0.7
VT34 *Glomus sp.*	0	0	0	0	0	0	0	3	0	3	0	0	3	0.0	0.0	1.5	1.1
VT35 *Dominikia sp.*	1	0	0	0	0	0	2	0	0	3	1	0	2	0.8	0.0	1.0	1.1
VT39 *Glomus sp.*	2	1	1	0	0	0	0	0	0	3	3	0	0	2.5	0.0	0.0	1.1
VT40 *Dominikia sp.*	3	0	2	0	0	0	0	0	0	3	3	0	0	2.5	0.0	0.0	1.1
VT50 *Microkamienskia sp.*	1	0	0	0	0	0	0	3	1	3	1	0	3	0.8	0.0	1.5	1.1
VT51 *Glomus sp.*	0	0	1	0	0	0	0	0	3	3	1	0	3	0.8	0.0	1.5	1.1
VT52 *Glomus sp.*	0	0	0	0	0	1	0	2	0	3	0	1	2	0.0	1.8	1.0	1.1
VT32 *Glomus sp.*	0	0	0	0	0	1	0	4	0	4	0	1	4	0.0	1.8	2.0	1.5
VT44 *Archaeospora sp.*	0	0	0	0	0	0	4	0	0	4	0	0	4	0.0	0.0	2.0	1.5
VT48 *Claroideoglomus sp.*	0	0	2	0	0	0	3	3	2	5	2	0	5	1.7	0.0	2.5	1.8
**VT36 *Dominikia sp.***	0	0	0	0	0	0	0	1	6	6	0	0	6	0.0	0.0	3.0	**2.2**
**VT45 *Archaeospora sp.***	0	0	0	0	0	3	0	4	0	6	0	3	4	0.0	5.4	2.0	**2.2**
**VT30 *Dominikia sp.***	6	1	3	0	0	0	0	0	0	7	7	0	0	5.9	0.0	0.0	**2.6**
**VT46 *Dominikia sp.***	0	0	1	0	0	0	8	0	3	8	1	0	8	0.8	0.0	4.1	**2.9**
**VT37 *Dominikia sp.***	0	0	0	0	0	0	0	9	0	9	0	0	9	0.0	0.0	4.6	**3.3**
**VT38 *Microdominikia sp.***	1	0	0	0	1	0	1	8	3	9	1	1	9	0.8	1.8	4.6	**3.3**
**VT42 *Dominikia sp.***	7	6	8	0	0	0	2	1	4	13	9	0	6	7.6	0.0	3.0	**4.8**
**VT47 *Entrophospora sp.***	11	7	8	1	3	8	10	4	2	17	15	8	10	12.6	14.3	5.1	**6.2**
Total OTUs:	80	49	69	3	10	58	146	91	153	273	119	56	197				

Note: the species with total rate of OTUs >1% are highlighted in bold.

Table S7. Identification of arbuscular mycorrhizal fungi: genera list according to OTUs founded by ITS2 analysis.

Species list for ITS2 / Number of OTUs for ITS2	OTUs for meadow biotopes			OTUs for forest biotopes			OTUs for river valley (grassland, disturbed area) biotopes			Total	Total OTUs for			Total % of OTUs for			Total %
	STP #1	STP #3	STP #4	STP #7	STP #8	STP #9	STP #11	STP #12	STP #13	OTUs	Meadow	Forest	Valley	Meadow	Forest	Valley	of OTUs
Dominikia	20	7	14	0	0	0	17	13	17	56	24	0	38	20.2	0.0	19.3	20.5
Rhizophagus	17	12	10	1	5	22	32	12	92	51	22	22	40	18.5	39.3	20.3	18.7
Glomus	7	5	7	1	0	4	3	12	5	27	14	5	16	11.8	8.9	8.1	9.9
Entrophospora	14	11	11	1	3	10	12	9	6	24	19	10	15	16.0	17.9	7.6	8.8
Claroideoglomus	12	8	10	0	0	3	37	21	18	23	12	4	20	10.1	7.1	10.2	8.4
Archaeospora	0	3	0	0	0	3	15	4	1	21	3	3	19	2.5	5.4	9.6	7.7
Acaulospora	2	1	11	0	0	4	6	0	0	19	13	4	6	10.9	7.1	3.0	7.0
Paraglomus	1	1	2	0	1	2	14	4	2	15	3	2	15	2.5	3.6	7.6	5.5
Funneliformis	0	0	0	0	0	0	7	2	0	9	0	0	9	0.0	0.0	4.6	3.3
Microdominikia	1	0	0	0	1	0	1	8	3	9	1	1	9	0.8	1.8	4.6	3.3
Ambispora	1	0	1	0	0	2	2	1	1	5	2	2	3	1.7	3.6	1.5	1.8
Scutellospora	0	0	2	0	0	7	0	2	0	4	1	2	2	0.8	3.6	1.0	1.5
Microkamienskia	1	0	0	0	0	0	0	3	1	3	1	0	3	0.8	0.0	1.5	1.1
Rhizoglomus	3	1	1	0	0	0	0	0	0	3	3	0	0	2.5	0.0	0.0	1.1
Diversispora	1	0	0	0	0	1	0	0	0	2	1	1	0	0.8	1.8	0.0	0.7
Cetraspora	0	0	0	0	0	0	0	0	1	1	0	0	1	0.0	0.0	0.5	0.4
Halonatospora	0	0	0	0	0	0	0	0	6	1	0	0	1	0.0	0.0	0.5	0.4
Total OTUs:	80	49	69	3	10	58	146	91	153	273	119	56	197				

Table S8. List of common and endemicspecies (except VT) ranked by decreasing number of OTUs.

Region	Meadow biotopes	Forest biotopes	River valley (grassland, disturbed area) biotopes
	**Common species**		
**ITS1**	*Rhizophagus intraradices, Dominikia bernensis, Paraglomus laccatum, Rhizophagus irregularis, Claroideoglomus claroideum, Glomus indicum, Septoglomus constrictum, Acaulospora paulinae, Glomus tetrastratosum, Septoglomus nigrum, Otospora bareae, Ambispora leptoticha, Glomus macrocarpum*		
**ITS2**	*Rhizophagus intraradices, Rhizophagus irregularis, Paraglomus laccatum, Claroideoglomus claroideum, Acaulospora paulinae, Entrophospora infrequens, Claroideoglomus lamellosum*		
	**Endemic species**		
**ITS1**	*Acaulospora alpina*<br>*Paraglomus brasilianum*<br>*Acaulospora viridis*	*Diversispora sporocarpia*<br>*Diversispora spurca*<br>*Diversispora slowinskiensis*	*Funneliformis mosseae*<br>*Septoglomus viscosum*<br>*Palaeospora spainii*<br>*Diversispora celata*<br>*Claroideoglomus lamellosum*<br>*Halonatospora pansihalos*<br>*Dominikia achra*<br>*Diversispora varaderana*<br>*Acaulospora delicata*
**ITS2**	*Acaulospora brasiliensis*<br>*Rhizoglomus melanus*<br>*Acaulospora alpina*<br>*Glomus bareae*<br>*Diversispora varaderana*	*Scutellospora alterata*	*Funneliformis mosseae*<br>*Rhizophagus aggregatus*<br>*Archaeospora spainiae*<br>*Ambispora fennica*<br>*Scutellospora pellucida*<br>*Halonatospora pansihalos*<br>*Dominikia difficilevidera*<br>*Claroideoglomus hanlinii*<br>*Cetraspora gilmorei*<br>*Acaulospora punctata*

Table S9. Observed and extrapolated values of species richness, Shannon diversity and Simpson diversity by OTUs.

Hill Number	Biotope	ITS1		ITS2	
		Observed	Estimated	Observed	Estimated
Species richness (q=0)	meadow	43.0	58.0[a]	35.0	55.2[a]
	forest	34.0	42.1[b]	20.0	38.0[a]
	valley	52.0	52.7[a]	45.0	46.5[a]
Shannon diversity (q=1)	meadow	13.1	13.4[b]	10.0	10.2[b]
	forest	13.6	14.3[a]	6.0	6.2[c]
	valley	14.2	14.2[a]	14.5	14.6[a]
Simpson diversity (q=2)	meadow	7.1	7.1[c]	6.1	6.1[b]
	forest	8.6	8.7[b]	3.8	3.8[c]
	valley	8.9	8.9[a]	8.3	8.3[a]

Note: Different letters ("a", "b", etc.) indicate significant differences within the same parameters (ANOVA and Tukey's test; $P < 0.05$).

Table S10. The advantages and disadvantages of molecular genetic methods using Illumina MiSeq to identify AMF species in comparison with morphological methods

Illumina MiSeq Advantages	Illumina MiSeq Disadvantages
1) The application of universal primers makes it possible to compare the diversity of not only the fungi belonging to the Glomeromycetes class, but also the fungi of different classes in one sample	1) The lack of full reference database of sequences (for example, the NCBI GenBank contains sequences of only 3 species – *P. laccatum* , *P. brasilianum* , *P. occultum* [31] of 9 known species of *Paraglomus* genus – *P. albidum* , *P. bolivianum* , *P. brasilianum* , *P. laccatum* , *P. lacteum* , *P. occidentale* , *P. occultum* , *P. pernambucanum* , *P. turpe* ; the MaarjAM database contains many sequences unidentified up to the species level)
2) The facility to identify AM fungi in the absence of fungal spores in the source soil that would be impossible through morphological analysis	2) The availability of multiple databases creates complexity in data processing and registration of results (NCBI, EMBL-EBI, DDBJ, MaarjAM, UNITE; [89])
	3) The presence of a significant number of sequences in the GenBank with incorrect identification (for example, [90])
3) The depth of coverage, i.e. the possibility of sequencing with high depth, that allows covering a significant part of AMF true polymorphism	4) NGS methods allow obtaining only short DNA fragments (up to 240 bp for ITS1 and up to 350 bp for ITS2 by Illumina MiSeq application; 170-750 bp with other NGS methods; [89]), appearance of chimeras
	5) Lack of clear boundaries for a number of species and genera of AM fungi due to different polymorphism in diverse regions of barcoding (different values of *p* -distance, see Figure S3)
